# A multiple filter-wrapper feature selection algorithm based on process optimization mechanism for high-dimensional omics data analysis

**DOI:** 10.1371/journal.pone.0338051

**Published:** 2025-12-11

**Authors:** Yongtao Shi, Yuefeng Zheng, Xiaotong Bai

**Affiliations:** School of Mathematics and Computer, Jilin Normal University, Siping, Jilin, China; Indiana University School of Medicine, UNITED STATES OF AMERICA

## Abstract

Recently, hybrid feature selection methods have demonstrated excellent performance on high-dimensional data, but many of these methods tend to yield relatively homogeneous feature subsets. To address this, we propose a novel hybrid feature selection algorithm called the Hybrid Multiple Filter-Wrapper algorithm. This algorithm employs a dual-module structure: Module 1 utilizes the random forest feature importance method to achieve significant dimensionality reduction of the original feature set, resulting in the candidate feature subset *F*_1_. In Module 2, we first propose a bivariate filter algorithm: the minimum Spearman-Maximum Mutual Information method. This method assesses both the correlation and redundancy of *F*_1_, whose results are then fed into the wrapper algorithm for further exploration. Furthermore, we integrate two swarm intelligence algorithms to develop the Hybrid Grey Wolf and Chaotic Dung Beetle Wrapper Algorithm. This algorithm incorporates chaos theory to enhance the position update mechanism of the Dung Beetle Algorithm, then embeds Dung Beetle Algorithm into the Grey Wolf Algorithm, thereby balancing exploration and exploitation capabilities. Finally, a process optimization mechanism based on the theory of random laser intensity fluctuations dynamically monitors the optimization process. Upon convergence of the wrapper algorithm to a local optimum, the filter algorithm is restarted, and chaos theory is used to reset the population. This process enhances the diversity of both the candidate feature subset and the population, effectively avoiding local optima. We extensively compare our method with ten hybrid algorithms from the past three years across ten public benchmark datasets from MGE. Experimental results show that our algorithm outperforms the most other algorithms: on all datasets, it achieves an average classification accuracy that is at 1.3% least higher, an average feature subset length that is at least 8 units shorter, and a dimensionality reduced to less than 0.45% of the original. The results are statistically significant.

## 1 Introduction

Over the past few decades, vast amounts of data has been generated across various fields [1]. These datasets often have high dimensionality and noise, which poses a significant challenge in removing irrelevant and redundant features for data classification [2,3].Therefore, feature selection has become a central focus for researchers aiming to reduce computational complexity, improve data analysis accuracy, and enhance operational efficiency [3].

Feature selection methods are categorized into filter [5] and wrapper approaches. Filter methods score features based on specific metrics, selecting those with the highest scores. While less computationally demanding, they often exhibit limited generalizability [6]. Some researchers have suggested filter algorithms that bypass initial screening and select features directly from the dataset, often leading to an overly broad and unsatisfactory selection [7,8].

Wrapper methods [9] combine optimization algorithms and classifiers to search the original feature set and generate candidate subsets. Classifiers then assess these subsets, typically achieving high classification accuracy (ACC). Common optimization algorithms include Genetic Algorithm (GA) [10], Particle Swarm Optimization (PSO) [11], Antlion Optimization (ALO) [12], and Cuckoo Search (CS) [13] among other swarm intelligence optimization algorithms. In 2022, researchers, including Xue et al., introduced the Dung Beetle Algorithm (DBA) [14], a novel algorithm with robust global search and parallel computing capabilities, although it lacks high solution accuracy [15]. Currently, DBA-based methods are primarily applied in engineering [16]. The superior global search and local exploration capabilities of swarm intelligence algorithms have led many researchers to apply them in feature selection [16,17]. Hybrid feature selection methods utilizing DBA are less developed compared to other swarm intelligence algorithms [17]. Therefore, this paper considers using the Dung beetle algorithm for Wrapper feature selection based on a hybrid swarm intelligence strategy.

In 2017, Jain et al. proposed a method combining univariate Filter and an improved PSO method for gene selection, achieving excellent classification performance [18]. However, the high dimensionality of genes and the limited number of samples complicate analysis and classification, and the BPSO algorithm tends to converge to local optima early, making it difficult to achieve the global best [18].In 2021, Wang et al. and Wojdan et al. introduced a hybrid filter-wrapper algorithm [19]. The filter method selects a subset of high-scoring features using specific metrics, effectively narrowing the search scope [20]. The wrapper method employs an optimization algorithm to search filtered feature subsets and selects the optimal one based on classification accuracy. While the hybrid algorithm combines the filter’s speed with the wrapper’s accuracy [21], it still struggles with local optima [22].

In 2022, Pashaei et al. ’s research combined Maximum Relevance Minimum Redundancy (mRMR) with the Binary Artificial Owl Optimization (BAO) algorithm, showing excellent performance. However, it performed poorly on some benchmark datasets, indicating the limitations of its mutation mechanism [23]. In 2023, the team used the recently compiled Turkey earthquake database to test a reliable and reasonable data-based CAV prediction model for seismic disaster analysis, structural health monitoring, geotechnical engineering investigations, and early earthquake warning analysis [24]. In 2024, Sosa et al. ’s research emphasized the importance of feature collaboration, showing that their combined contribution provides more information about category labels than individual features themselves [25].

In 2024, Lawrence et al. developed an efficient feature selection and classification method for high-dimensional microarray cancer data, noting that this data faces a strong “curse of dimensionality,” and the computational power required may limit the effectiveness of existing methods [26]. In the same year, Dr. José introduced the Iterative Linear Association Analysis method, which can effectively observe multicollinearity between variables and generate more accurate explanations using linear modeling techniques for result prediction [27]. In 2025, Pashaei et al. developed a multi-filter gene selection method to identify biomarkers for Alzheimer’s disease by integrating various ranking techniques and feature selection algorithms to improve accuracy [28]. In the same year, Pashaei et al. introduced two novel hybrid gene selection techniques, which improved search behavior by integrating crossover and mutation operators [29]. However, the algorithm may require longer execution times, especially when processing very large datasets. Additionally, high-dimensional, heterogeneous data poses challenges to Filter methods, limiting their effectiveness [29].

In summary, current research faces three main challenges: 1) Univariate Filter methods struggle with the theoretical limitation of feature interaction compensation, while bivariate Filter methods are challenged by both the curse of high dimensionality and limited sample sizes; 2) The literature has not fully explored the collaborative framework between the DBA and the grey wolf algorithm (GWA); 3) There is limited research on interdisciplinary regulatory methods in hybrid feature selection. Additionally, most existing hybrid algorithms lack dynamic interaction between the filter and wrapper stages, and the wrapper algorithm is susceptible to local optima due to poor initial feature quality.

To address these challenges, this study first combines univariate and proposed bivariate Filter algorithms in a stepwise manner, effectively addressing the issues of high dimensionality and limited sample sizes in omics data. Next, improvements are made to two swarm intelligence algorithms to enhance the strengths of each, filling the gap in research on hybrid swarm intelligence algorithms based on the DBA. Finally, an interdisciplinary algorithm regulation method, combining physics and computer science, is designed based on a physical optics model. The result is the development of a dual-module hybrid feature selection algorithm, named Hybrid Multiple Filter-Wrapper (HMF-W).

In this approach, the M1 module uses the Random Forest-based Feature Importance Method (RF-FIM) [39] for significant dimensionality reduction of the original feature set, resulting in a candidate feature subset. The M2 module integrates the minimum Spearman Maximum Mutual Information (mSMMI) with the Hybrid Gray Wolf and Chaotic Dung Beetle Wrapper (HGW-CDBW) algorithms, using the proposed interdisciplinary algorithm control method, the Process Optimization Mechanism (POM), to further reduce and optimize the candidate feature subset. The main contributions of the HMF-W algorithm are introduced in three aspects:

(1) This study proposes a nonlinear bivariate Filter algorithm, the mSMMI algorithm. It measures the correlation between features and labels, as well as feature redundancy in the candidate subset, using mutual information and Spearman’s rank correlation coefficient. Two parameters are designed to adjust evaluation weights for correlation and redundancy, enabling feature measurement from multiple perspectives. Additionally, RF-FIM is used for significant dimensionality reduction, followed by the mSMMI algorithm, which selects features from multiple perspectives in sequence, overcoming the bottleneck of feature interaction compensation and reducing the feature dimensions for both the mSMMI and subsequent Wrapper algorithms.

(2) We improve and combine two swarm intelligence algorithms to form the Wrapper algorithm, HGW-CDBW. This algorithm uses chaotic disturbances to control the movement direction and step size of dung beetle individuals, enhancing the first three optimal solution areas of the gray wolf algorithm. It combines the GWA’s excellent global search ability with the dung beetle algorithm’s enhanced local development capability, enabling HGW-CDBW to maintain a balance between exploration and exploitation, achieving the goal of selecting the optimal feature subset.

(3) We combine the theory of random laser intensity fluctuations with the proposed average improvement rate and extreme value control strategy to form the POM mechanism. When the Wrapper algorithm falls into local optima, it dynamically adjusts the execution frequency of the bivariate Filter and Wrapper algorithms, using the results of the bivariate Filter algorithm to initialize the population with a Logistic chaotic representation. This increases the diversity of the candidate feature subset and the population, helping the algorithm to escape local optima and find the optimal feature subset.

The rest of this paper is organized as follows: Sect 2 reviews related work. Sect 3 introduces the proposed algorithm. Sect 4 presents experimental studies and result analysis, followed by a conclusion in Sect 5.

## 2 Related works

In this section, we will introduce seven important theories and methods, namely random laser intensity fluctuation theory, chaos theory, random forest-based feature importance methods, GWA algorithm, and DBA algorithm. These theories lay a solid foundation for the HMF-W algorithm.

### 2.1 The random lasers intensity fluctuations theory

Random Laser Intensity Fluctuations (RLIF) theory, discussed by Anderson S.L. Gomes and others, explores the fluctuations in light intensity caused by multiple reflections and interference in random Laser without fixed cavities and random scattering media [30]. It suggests that the probability density function of laser intensity is represented by an exponentially truncated Lévy-like distribution, which depicts a wide range of intensity fluctuations [31]. In such a distribution, as the input excitation power increases, the accumulation of electromagnetic energy also increases, thereby raising the tail index κ of the intensity distribution [32]. This means the fluctuations in intensity are quite significant, and the intensity distribution tends toward a Gaussian distribution, due to the Central Limit Theorem [30]. Additionally, the collective effect of pseudo-modes in random Laser is considered. Due to the varying contributions from each scattering center, the interference patterns of light waves exhibit high randomness and irregularity, which are characteristic of the intensity fluctuations in random Laser [31]. Random laser intensity fluctuations can be expressed as the superposition of multiple independent scattering events, each of which can be represented by a sine wave [32]. The mathematical formula is as follows:

$$I\left(t\right)=I_{0}+\sum_{i=1}^{N}A_{i}\sin\left(2\pi f_{i}+\varphi_{i}\right)$$ (1)

where, *I*_0_ represents the average light intensity, while $A_{i},f_{i}$ and $\varphi_{i}$ denote the amplitude, frequency, and phase of the i-th sine wave, respectively [33].

### 2.2 Chaos theory

Chaos theory studies pseudo-random, unpredictable behaviors in deterministic nonlinear systems, with its core being the system’s extreme sensitivity to initial conditions (the butterfly effect) and inherent pseudo-randomness [34]. This pseudo-randomness is not true randomness, but rather a sequence generated by deterministic equations, which is ergodic and non-periodic [35]. Chaotic systems effectively represent high-dimensional, nonlinear, strongly coupled iterative processes, similar to the search trajectory of swarm intelligence algorithms [36]. When the system’s state deviates from a stable path due to parameter disturbances or boundary condition changes, the chaotic mechanism quantifies its fluctuations, revealing hidden patterns not captured by traditional linear analysis [37]. This paper uses the Logistic map to generate chaotic sequences for discrete systems, with the formula as follows:

$$x_{n+1}=Seed\cdot x_{n}\cdot \left(1-x_{n}\right)$$ (2)

where, Seed is the chaotic seed within the range (3,4), and in this paper, it is set to 3.9, where *x*_*n*_ is the current value and *x*_*n*_ + 1 is the next value. At this point, the system enters chaos, showing extreme sensitivity to initial values [38].

Firstly, rounding errors inevitably occur when initializing the population in a wrapper algorithm, leading to significant deviations in long-term behavior. The algorithm then searches the solution space diffusely, making it difficult to concentrate locally. However, chaotic sequences can infinitely approach any point within a finite range, offering more efficient search space coverage than uniform distribution. Therefore, this paper considers using chaos theory to improve the population initialization part. Secondly, chaotic sequences have an almost non-repetitive characteristic. They can inject continuous diversity into the algorithm, suppressing premature convergence. Thus, this paper considers guiding the positions of individual agents in swarm intelligence algorithms through chaotic sequences to enhance their development ability in smaller feature spaces.

### 2.3 RF-FIM

The feature importance in random forests is calculated by adding the gains from features at each node across all decision trees. entropy of information is often used to measure feature contributions in classification problems [39].

$$H(D)=-\sum_{j=1}^{\left|C\right|}\frac{\left|D_{j}\right|}{\left|D\right|}\log_{2}\left(\frac{\left|D_{j}\right|}{\left|D\right|}\right)$$ (3)

$$IG=H(D)-\sum_{i=1}^{\left|S\right|}\frac{\left|D_{i}\right|}{\left|D\right|}H(Di)$$ (4)

where, H(D) is the Entropy of the dataset D, C is the Category set, *D*_*j*_ is the Subset of the category j, H(D|S) is the Conditional Entropy of after given a subset of feature , |S| is the size of the feature subset, |D| is the size of the dataset. The contribution of each decision tree to feature importance is the average reduction in information entropy of that feature across all trees [40]. Feature importance evaluation in random forests is done through the contribution of node splits in the integrated decision trees, providing quantifiable screening criteria for high-dimensional features. Its advantage is that it can handle nonlinear relationships without requiring independent feature distribution assumptions [41].

The purpose of using the RF-FIM algorithm in the feature selection process is to select a small number of features with high information gain from the original dataset. This helps reduce the complexity of model training and improves efficiency [42]. The number of selected features is based on the total number of features in the dataset and depends on the size of the dataset and samples.

### 2.4 The gray wolf optimization algorithm

As a typical swarm intelligence algorithm, the Grey Wolf algorithm (GWA) [43] simulates and optimizes the social hierarchy and group hunting behaviors of Grey Wolf populations. The gray wolf population has a strict social hierarchy, categorized into four types: *α* wolves, *β* wolves, *δ* wolves, and *ω* wolves, with lower ranks having more wolves. *ω* Grey Wolf individuals *X*_*i*_ in the pack update their positions based on the locations of *α* wolf, *β* wolf, and *δ* wolf, $X_{\alpha }$, $X_{\beta }$, $X_{\delta }$ [43], with the update formula as follows:

$$D_{\alpha }=\left|C_{\text{1}}\times \;X_{\alpha }\left(t\right)-X_{i}\left(t\right)\right|$$ (5)

$$D_{\beta }=\left|C_{\text{2}}\times \;X_{\beta }\left(t\right)-X_{i}\left(t\right)\right|$$ (6)

$$D_{\delta }=\left|C_{\text{3}}\times \;X_{\delta }\left(t\right)-X_{i}\left(t\right)\right|$$ (7)

$$X_{1}=X_{\alpha }(t)-A_{1}\times D_{\alpha }$$ (8)

$$X_{\text{2}}=X_{\beta }(t)-A_{\text{2}}\times D_{\beta }$$ (9)

$$X_{\text{3}}=X_{\delta }(t)-A_{\text{3}}\times D_{\delta }$$ (10)

$$X_{i}(t+1)=\frac{X_{1}(t+1)+X_{2}(t+1)+X_{3}(t+1)}{3}$$ (11)

Where, D represents the distance between the grey wolf and its prey, and t represents the iteration. X and *X*_*p*_ represent the position vectors of the Grey Wolf and the prey, while A and C are vector coefficients, with their specific mathematical expressions given by formulas (8) and (9) [43]:

$$A=2\times a\times r_{1}-a$$ (12)

$$C=2\times r_{2}$$ (13)

Where, a is the convergence factor, *r*_1_ and *r*_2_ are random vectors taking values in [0,1]. As the number of iterations increases, the value of a gradually decreases from 2 to 0 [44].

When handling feature selection problems, the GWA algorithm needs to be converted into a binary form to facilitate feature selection. However, existing methods of binary feature selection usually rely on random number comparisons, making it difficult to avoid selecting all or no features. While the GWA algorithm has excellent global search capabilities, it tends to get trapped in local optima in the later stages of iteration. Therefore, this paper first considers adopting a more robust approach to convert continuous algorithms into discrete ones, and then applies a strategy that combines two swarm intelligence optimization algorithms to improve the grey wolf algorithm, forming a new algorithm that balances global search and local development as part of the wrapper.

### 2.5 Dung beetle algorithm

**Fig 1:**
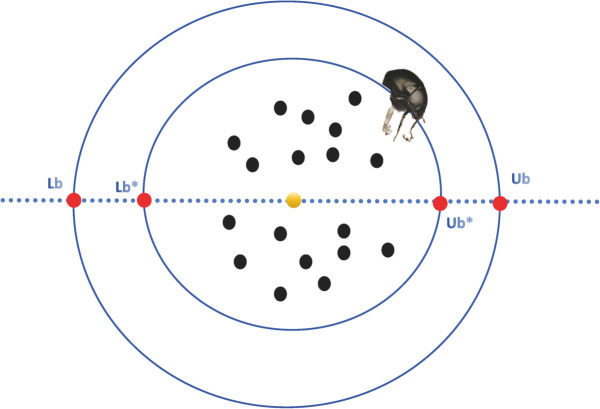
Strategy of Dung Beetle’s boundary selection. Conceptual model of Dung Beetle’s boundary selection strategy.

The Dung Beetle Algorithm (DBA) [14] is a population based swarm intelligence optimization algorithm proposed in 2022. This algorithm is mainly influenced by the behaviors of dung beetles such as rolling, reproduction, foraging, and stealing, with the dung beetle population divided into different roles in a 6:6:7:11 ratio [14]. DBA has advantages in convergence, search effectiveness, and exploration capabilities, but some phases of behavior that develop around local optima often remain at local optima [14], The following Fig 1. shows the conceptual model of the dung beetle’s boundary selection strategy [16].

The foraging behavior of dung beetles selects safe areas, and the foraging area for larvae dynamically updates [14], represented equation as follows:

$$\begin{array}{c} Lb^{b}=\max(X^{b}\times (1-R),Lb)\\ Ub^{b}=\min(X^{b}\times (1+R),Ub) \end{array}$$ (14)

Here, *X*_*b*_ is the local optimal position of the current population, *Lb^b^* and *Ub^b^* define the lower and upper bounds of the optimal foraging area [16], The position update equation for the foraging dung beetles is as follows:

$$x_{i}(t+1)=x_{i}(t)+{\text{Y}}_{1}\times (x_{i}(t)-Lb^{b})+{\text{Y}}_{2}\times (x_{i}(t)-Ub^{b})$$ (15)

Where *Y*_1_ is a random number following a normal distribution, and *Y*_2_ is a random vector between 0 and 1, with a size of 1 × Dim [16].

Although DBA has a unique boundary selection strategy, it struggles with exploring the solution space. Since the optimal search in each iteration is updated around the optimal individual, the position of the optimal individual is crucial. The original DBA algorithm struggles to deeply analyze the optimal individual, leading to some waste in the utilization of the optimal individual.

To improve DBA’s ability to explore neighborhood optima and solve the premature convergence problem, this study first considers introducing the chaos theory introduced in Sect 2.2 to guide the position of dung beetle individuals, hoping to further enhance DBA’s local development ability. Then, it considers adopting a unique embedded structure that combines the improved DBA and the GWA algorithm introduced in the previous section, which can minimize the premature convergence issue. This forms a novel wrapper feature selection algorithm based on a hybrid swarm intelligence algorithm.

### 2.6 Previous hybrid feature selection methods

In the field of feature selection, previous researchers combined the advantages of filter and wrapper methods to develop many excellent hybrid algorithms.They have made significant contributions to the research field. The following Table 1 summarizes the five hybrid feature selection algorithms from 2021 to 2024.

**Table 1:** Recent hybrid feature selection methods.

Authors	Year	Algorithm	Brief description	Limitations
Got [47]	2021	FW-GPAWOA	Multi-objective optimize	Few comparable methods and test datasets
Zhu [48]	2022	HFSIA	Mutation and cauchy operator	High redundancy and computational complexity
Xu [49]	2023	FG-HFS	The markov blankets	Requires manually set thresholds, ignoring redundancy between features. Easy to local optima
Deng [50]	2023	FTGGA	Adaptive threshold strategy	Convergence is slow. Easy to generate poor solutions
Li [51]	2024	SIFFS	Define similar fuzzy space	Incomplete search of solution space. Easy to local optima

In 2021, Got et al. attempted to merge filter and wrapper methods, using Whale Optimization Algorithm (WOA) to address the multi-objective nature of feature selection [47]. However, multi-objective optimization remained at the algorithm level, with experimental validation only designed using 2 multi-objective methods and 3 datasets [47]. This makes the results difficult to generalize. In 2022, Zhu et al. proposed an HFSIA algorithm to address the challenges of high-dimensional data. The algorithm combines filter methods and introduces a deadly mutation mechanism and Cauchy operator to enhance algorithm performance for dimensionality reduction [48]. However, the high redundancy of features led to poor classifier performance. At the same time, its fitness evaluation method was inefficient, leading to high computational complexity.

In 2023, Xu et al. discussed the method of using Markov blankets for feature selection, focusing on the relationship between features and classes [49]. They emphasized computing symmetrical uncertainty to evaluate the shared information between features and classes, helping to identify relevant features. Meanwhile, the study acknowledged the limitations of the datasets used. Secondly, the algorithm is prone to local optima due to neglecting feature interactions. Lastly, manually set thresholds are required, making it difficult to find the global optimal solution. In the same year, Deng et al. proposed a strategy involved continuously updating the feature threshold set to guide the evolution of genetic algorithms, enhancing search capability [50]. However, the unguided crossover and mutation in the genetic algorithm led to excessive randomness and the generation of poor solutions. Its randomness causes slow convergence of the algorithm.

In 2024, Li et al. designed a heuristic search based forward feature selection algorithm to enhance the exploration of feature subsets [51]. However, the cooperative swarm intelligence feature selection algorithm has limitations in searching the entire solution space, making it easy to overlook better solutions. Secondly, the designed algorithm often sacrifices the globally optimal feature combination to meet output conditions. Lastly, the heuristic algorithm suffers from early termination, leading to repeated feature subsets and invalid features.

Previous researchers’ work demonstrated the optimization potential of hybrid feature selection but still had the following shortcomings:

1) Static thresholds or fixed output conditions are difficult to adapt to changes in data distribution.

2) Most methods focus on classification accuracy, ignoring multi-objective collaborative optimization of feature subset length and feature redundancy.

3) Meta-heuristic methods have low computational efficiency in ultra-large feature spaces, leading to a surge in computational costs.

To address these issues, the proposed HMF-W algorithm first uses an extremum control strategy in the proposed process optimization mechanism to adaptively adjust the threshold, thus accommodating real-time changes in the optimization process. Next, the proposed algorithm uses an innovative algorithmic framework, where the univariate Filter method is responsible for significant dimensionality reduction. The POM mechanism regulates the alternating execution of the bivariate Filter method and the improved wrapper method to repeatedly optimize the result feature subset of the univariate Filter method. This method not only ensures a high ACC while selecting shorter feature subsets, but also significantly reduces computational complexity when dealing with high-dimensional data.

## 3 Hybrid multiple filter-wrapper algorithm

In this section, we will provide a detailed introduction to the POM-based Hybrid Multiple Filter-Wrapper(HMF-W) algorithm. The algorithm consists of two modules: Module 1 consists of the RF-FIM algorithm; Module 2 consists of the Filter mSMMI algorithm, the wrapper HGW-CDBW algorithm, and the process optimization mechanism.

In Module M1, we use the RF-FIM algorithm mentioned in Sect 2.3, which measures the contribution of features based on the random forest method. We treat it as feature importance and use it as a criterion to select a set of important features from the original dataset for further dimensionality reduction by other algorithms in M2, such as the nonlinear bivariate Filter mSMMI algorithm. This approach first significantly reduces the dimensionality of the original feature set, effectively reducing the length of the final feature subset. It then allows feature selection from three aspects: random forest, mutual information, and Spearman’s coefficient, preventing the omission of valuable features. Below, we detail the M2 module:

### 3.1 mSMMI filter algorithm

Feature selection algorithms, specifically filter algorithms, rank features in a dataset using specific metrics. These include univariate and multivariate methods, such as deep learning based feature selection and LASSO regression. LASSO regression performs feature selection using L1 regularization, which ensures sparsity in high-dimensional data. However, it relies on linear assumptions and is limited in handling nonlinear relationships.

Based on the renowned multivariate mRMR concept, to better consider both relevance and redundancy simultaneously, this paper introduces a new filter algorithm, mSMMI, which uses Maximum Mutual Information to measure the relevance between features and labels, and minimum Spearman to measure redundancy among features, dynamically adjusting the ratio of mutual information score to redundancy using two parameters, s and y.

#### 3.1.1 Maximum Mutual Information (MMI).

Mutual Information (MI) measures the dependency between two variables, indicating how much information about one variable can be obtained from the value of another. Traditional methods of measuring relevance, such as the Pearson correlation coefficient, can only capture linear relationships. Mutual information can measure complex nonlinear relationships. This makes mutual information extremely robust when dealing with datasets that have highly complex structures.

For two random variables X(*x*_1_, *x*_2_, ..., *x*_*n*_) and Y(*y*_1_, *y*_2_, ..., *y*_*n*_), mutual information MI(X;Y) is defined as:

$$MI(X;Y)=\sum_{x,y}p(x,y)\log\left(\frac{p(x,y)}{p(x)p(y)}\right)$$ (16)

Where, p (x, y) is the joint probability distribution of X and Y, and p(x) and p(y) are the marginal probability distributions of X and Y, respectively.

The value of mutual information can be interpreted as how much information about variable Y can be obtained if we know the value of variable X (and vice versa). The higher the value, the stronger the dependency between the two variables. If the mutual information is zero, it indicates that the two variables are independent.

**Fig 2:**
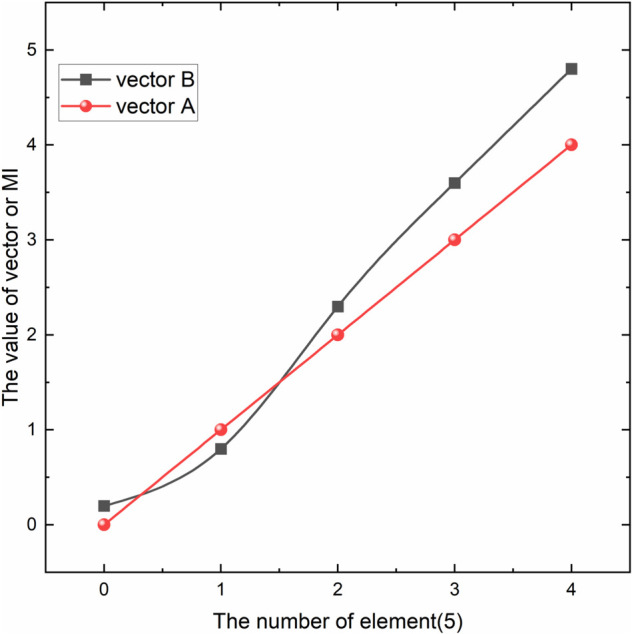
Relationship between vectors a and b. Nonlinear Relationship between vectors a and b.

**Fig 3:**
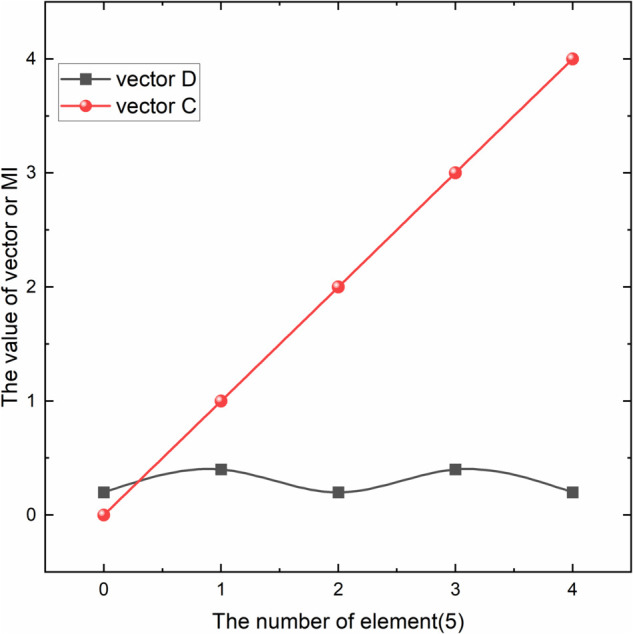
Relationship between vectors c and d. Weak positive correlation between vectors c and d.

Suppose we have two vectors a and b,where a = {0,1,2,3,4}, b = {0.2,0.8,2.3,3.6,4.8}. Using Eq (13), we calculate MI(a, b) = 1.609. The high mutual information indicates a strong nonlinear relationship between a and b, as shown in Fig 2. If the two vectors are c = {0,1,2,3,4} and d = {0.2,0.4,0.2,0.4,0.2}, using Eq (13), we calculate MI(a, b) = 0.673. The low mutual information indicates a weak positive correlation between c and d, as shown in Fig 3.

In high-dimensional raw datasets, there are thousands of features, which are reduced to hundreds of features by the M1 module to form a feature subset $F_{object}=\{f_{1},f_{2},...,f_{i},...,f_{n}\},i=1,2,...,n.$ Here, the feature *f*_*i*_ with the highest relevance to the label L is calculated using Eq (17):

$$MMI(F_{\text{object}},L)=MAX(MI(L,f_{i}))$$ (17)

where, MMI(*F*_*object*_, L) represents the maximum mutual information value between features and the label, *F*_*object*_ denotes the feature subset obtained after dimensionality reduction by module M1, L represents the label vector in the dataset, MAX is the maximum function, MI(L, *f*_*i*_) is calculated by Eq (16). and *f*_*i*_ denotes the features in *F*_*object*_, where i = 1, 2, ..., n.

#### 3.1.2 Minimum Spearman (mS).

In feature selection, the concept of maximum relevance and minimum redundancy considers both the correlation between features and labels and the redundancy among features.

The Spearman coefficient [38] is primarily used to measure the monotonic relationship between two variables, where one variable increases or decreases as the other does. The Spearman coefficient is less sensitive to the distribution and form of data, focusing more on the ordinal relationships between variables. This allows it to be used in analyzing both linear and non-linear monotonic relationships, making it more effective in handling non-normal distributions or data with outliers.

For any two features in the feature set $F_{object}=\{f_{1},f_{2},...,f_{i},...,f_{j},...,f_{n}\}$, denoted as *f*_*i*_ and *f*_*j*_, their Spearman coefficient is calculated by Eq (18).

$$\rho (f_{i},f_{j})=1-\frac{6\sum d_{i\text{j}}^{2}}{n(n^{2}-1)}$$ (18)

where, the Spearman coefficient between feature $\rho (f_{i},f_{j})\in$[-1,1], n is the number of features, *d*_*ij*_ is the difference in observation sequences of features *f*_*i*_ and *f*_*j*_,*i* = 1,2,...,*n*.

Given two features *F*_1_(0.8,0.8,0.7,0.4,0.9,0.6,0.7), *F*_2_(0.5,0,0,0.1,0.5,0.1,0.1), the Spearman coefficient $\rho (F_{1},F_{2})$=0.384, indicating a certain monotonic relationship between the two features, though it is relatively weak.

Eq (18) calculates the Spearman coefficient between known features and each feature in the set. Known features are denoted as *f*_*know*_, and a set of candidate features are denoted as $F_{\text{object}}=\{f_{1},f_{2},...,f_{k},...,f_{n-1}\},k=1,2,...,n-1,$ The feature *f*_*k*_ with the smallest Spearman coefficient value with the known features in the set is represented by Eq (19):

$$mS1(f_{\text{know}},F_{\text{object}})=MIN(\rho (f_{\text{know}},f_{k}))$$ (19)

Where, *f*_*know*_ denotes a Known feature, currently *f*_1_. *F*_*object*_ is the target feature set, at this point *F*_*object*−*know*_, and $mS1(f_{know},F_{object})$ denotes the minimum Spearman coefficient between the known feature and the target features set. The MIN function denotes the minimum value, *f*_*k*_ is a feature in *F*_*object*_, and the Spearman coefficient between *f*_*k*_ and *f*_*k*_*now* is the smallest within *F*_*object*_. The value of $\rho (f_{know},f_{k})$ is calculated using Eq (18).

The minimum Spearman (mS) between two feature sets is calculated as follows: the selected feature set is represented as *P*={p_1, p_2,..., p_i,..., p_m}, i=1,2,,…,m And the target set is denoted as $F_{\text{object}}=\{f_{1},f_{2},...,f_{n-m}\}.$ A feature is selected from the target set to satisfy the requirement that the Spearman coefficient is the minimum value between the selected feature and the known feature subset.The Spearman coefficient between the two feature sets is represented by Eq (20).

$$mS\text{2}(P,F_{\text{object}})=MIN(mS1(f_{\min},P))$$ (20)

Where, P is the selected feature set, *F*_*object*_ is the target feature set, *mS*2(*P*,*F*_*object*_) is the minimum Spearman coefficient between the target and selected feature sets. MIN is the minimum value function, *f*_*min*_ is the feature from *F*_*object*_ with the smallest Spearman coefficient, *mS*1(*f*_*min*_,*P*) calculated by Eq (19).

#### 3.1.3 Minimum Spearman Maximum Mutual Information (mSMMI).

In the M1 module, the RF-FIM algorithm reduces the number of features, cutting thousands down to a smaller set of hundreds with high information gain. In the M2 module, the bivariate filter algorithm further improves the selection, picking dozens of features that have high relevance and low redundancy. The number of features selected by the RF-FIM and mSMMI algorithms depends on the number of features and samples in the dataset, as well as the adjustment factor p and the mapping function f(x). This helps determine how many features will be selected by both algorithms. The formula for the number of features k2 selected by filter is:

$$K2=RandnumA+\frac{f}{rand()\;e}\cdot \cos(p\cdot e)$$ (21)

Where, the random number RandnumA, as the baseline, can provide a certain number of features for the filter algorithm. rand()∈(1,10), p∈(0,1) with p set to 0.8 in this case. where f and e represent the number of features and samples in the dataset, respectively. The cosine function term can decide the number of features to select based on the sample size of different datasets. In larger datasets, more diverse features can be selected to avoid an overly concentrated distribution of feature numbers across datasets. The number of features K1 selected by the RF-FIM algorithm is also calculated using Eq (21). where the range of the baseline RandnumA is expanded by 10 times. This allows for more high quality features to be provided for multiple runs of the mSMMI algorithm’s feature selection, with the rest remaining unchanged.

**Fig 4:**
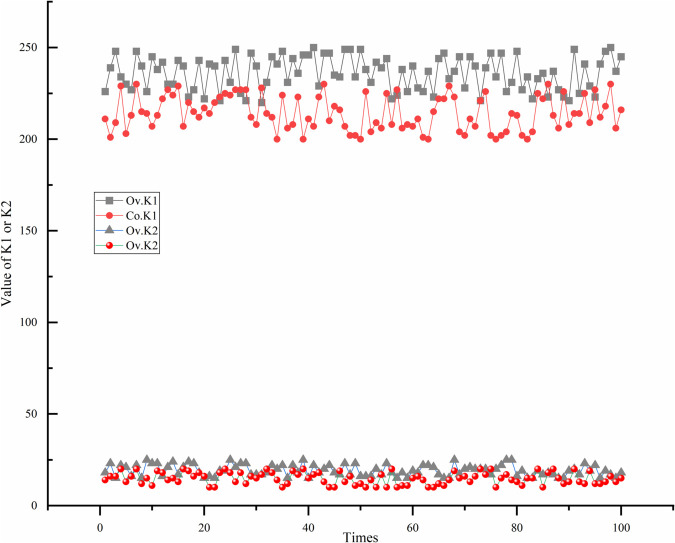
Evolution of K1 and K2 on the Co and Ov datasets. The trend of K1 and K2 values in the Co and Ov datasets.

Taking the Co and Ov datasets as examples, we use Eq (21) to calculate 100 iterations, and the variation of K1 and K2 values is shown in Fig 4 below.

From Fig 4, it can be seen that the K1 and K2 values obtained from Eq (21) in the Ov dataset are significantly greater than those obtained in the Co dataset. This is because the Ov dataset has more features and samples than the Co dataset. Therefore, on different datasets, the filter algorithm can select an appropriate number of high quality features using Eq (21).

We combine the previously mentioned maximum mutual information score and minimum Spearman coefficient to select features. After formulating MMI and mS into a single equation, we opt for subtraction to reduce computation. Subsequently, we introduce parameters into by Eq (22) to adjust the influence weights of mutual information scores and redundancy scores, thereby avoiding the situation where the influence weights of correlation and redundancy remain constant during the filter algorithm. In this way, we can select features one by one from the candidate feature subset.

$$mSMMI(p_{i})=\{\begin{array}{c} {}*20cs\cdot MMI(F_{\text{object}},L),i=0\\ s\cdot MMI(F_{\text{object}},L)-y\cdot mS1(p_{1},F_{\text{object}}),i=1\\ s\cdot MMI(F_{\text{object}},L)-y\cdot mS2(P,F_{\text{object}}),i>1 \end{array}$$ (22)

Where, s and y are iteratively generated based on Eqs (23) and (24), *F*_*object*_ as the candidate feature set, L is the label vector in the feature subset, P is the selected feature set, *p*_1_ is the first selected feature, and i denotes the number of selected features.

This study uses the mSMMI algorithm to find the best feature combination, which is then improved by the HGW-CDBW wrapper algorithm for precise feature selection. We use parameters s and y to adjust the influence of relevance and redundancy. Since the values of MMI and mS differ significantly across different datasets, the values of s and y cannot be 1. The value of s decreases while y increases, and they are calculated using Eqs (23) and (24).

$$s=\cos(\frac{i}{I}\cdot \frac{\pi }{2})$$ (23)

$$y=\sin(\frac{i}{I}\cdot \frac{\pi }{2})$$ (24)

**Fig 5:**
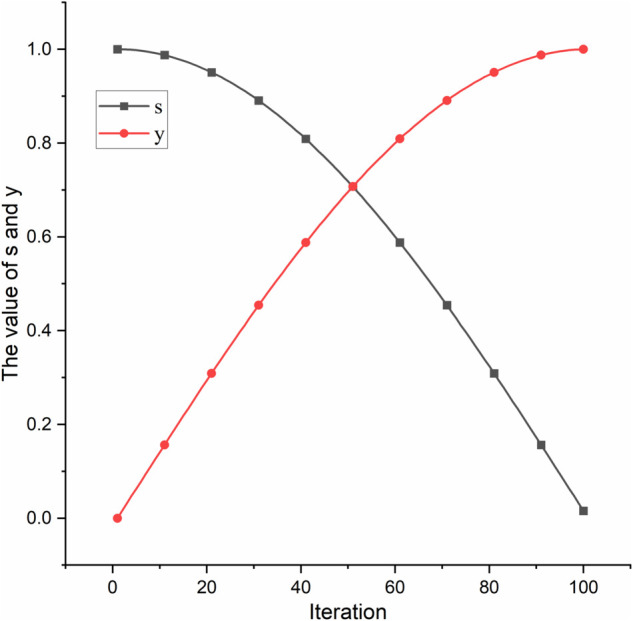
Trend of s and y. During the maximum number of iterations, s decreases from 1 to 0, and y increases from 0 to 1.

**Fig 6:**
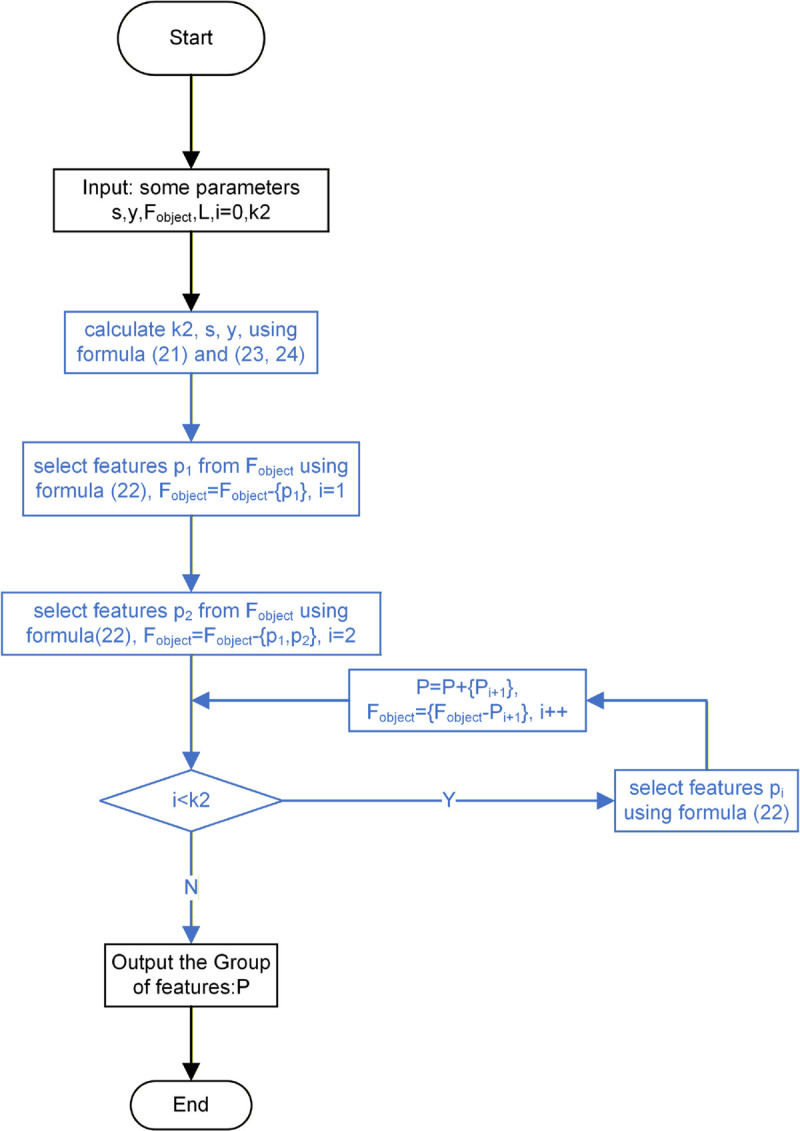
Flowchart of mSMMI algorithm. This flowchart is an introduction to the proposed filter algorithm mSMMI.

Where i is the current iteration of the wrapper algorithm, and I is the maximum number of iterations. s decreases from 1 to 0, and y increases from 0 to 1 during the wrapper algorithm’s iterations, as shown in Figs 5 and 6 shows the flowchart of mSMMI. Below is the pseudocode:

**Algorithm 1 mSMMI Algorithm Pseudocode.**



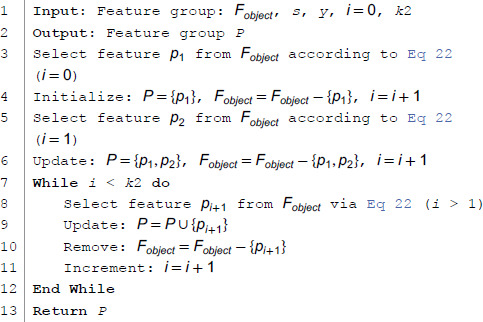



### 3.2 The hybrid gray wolf and chaotic dung beetle wrapper algorithm

To synergize the global convergence of the gray wolf optimization algorithm and the local exploitation advantages of the DBA algorithm, while avoiding the premature convergence flaw of DBA, this study improves the dung beetle and gray wolf optimization algorithms through a hybrid algorithmic strategy. Specifically, it combines the fine development ability of the chaotic dung beetle algorithm (CDBA) with the hierarchical leadership structure of the binary gray wolf algorithm. It then combines with a support vector machine classifier to form the Hybrid Gray Wolf and Chaotic Dung Beetle Wrapper (HGW-CDBW) algorithm.

#### 3.2.1 Chaotic dung beetle algorithm.

The Dung Beetle algorithm is inspired by the behavior of dung beetles, which use their antennae to sense their surrounding environment and push spherical dung to find the best burial spot. The algorithm solves optimization problems by simulating this behavior. This paper applies it to hybrid feature selection, using average ACC as the fitness function to evaluate the performance of feature subsets and find the optimal subset.

The dung beetle primarily searches around locally optimal solutions. However, this search is too blind and singular, often neglecting the exploration of nearby areas while approaching locally optimal solutions, reducing the versatility of the individual and frequently resulting in settling at local optima. This paper introduces chaos sequences into the dung beetle search process to increase chaotic disturbances, using Logistic mapping to generate chaos sequences, with the specific formula as follows:

$$\delta =Seed\cdot x_{n}\cdot \left(1-x_{n}\right)$$ (25)

Where, Seed is a chaotic seed with a value range of (3,4), set to 3.9 in this paper. *x*_*n*_ is the current value, *δ* is the next value in the chaotic sequence. n is the number of search agents, set to 30 in this paper.

One weakness of traditional DBA is its poor exploration of solution spaces, as each iteration optimally searches around the best individual. Thus the position of the best individual is crucial, but traditional DBA fails to analyze the best individual in depth, leading to some waste of the best individual’s utility.

To enhance DBA’s capability to explore local optima and address premature convergence, we employed chaotic sequences as the adjustment factor for search steps, achieving adaptability and diversity in the search process. Compared to the traditional DBA algorithm, the introduction of chaotic mapping increases the randomness and exhaustiveness of the search, allowing for more effective local exploration within the feature space while maintaining diversity and convergence in the search. In this paper, the initial position of the dung beetles is determined by the top three optimal solutions from the grey wolf algorithm. The following is the formula for the improved dung beetle position update:

$$x_{i}^{new}=x_{i}+\delta \cdot \text{sign}(x_{best}-x_{i})\cdot \log(1+|x_{best}-x_{i}|)$$ (26)

Where, *x*_*i*_ is the current position of the dung beetle, $x_{i}^{n}ew$is the new dung beetle position, and i is the individual dung beetle’s index number. *X*_*b*_*est* is the known best position, and *δ* is the step length factor generated based on Eq (25).

Each dung beetle having an independent step length factor. The sign function determines the direction of movement, and the log function adjusts the movement step length, decreasing as the distance increases, allowing for a precise approach to the optimal solution.

The introduction of chaotic theory aims to increase the diversity and local exploitation ability of the algorithm, avoiding premature convergence in CDBA algorithm and exploring more potential optimization areas. Below is the pseudocode for the CDBA algorithm:

**Algorithm 2 CDBA algorithm pseudocode.**



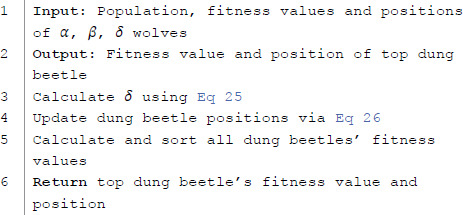



#### 3.2.2 Embedded dung beetle binary grey wolf algorithm.

In the discrete feature selection problem, each position vector dimension in the binary grey wolf algorithm corresponds to the selection status of a feature, where 1 indicates the feature is selected, and 0 means it is not selected. Position selection is usually assisted by random numbers, with the threshold generally fixed at 0.5. Although this position update method uses randomization to determine feature selection, it may result in cases where all random numbers are greater than or less than 0.5, leading to the selection of all features or no features being selected. To overcome it, we use a set of random numbers in ascending order, selecting the median from the sequence as a threshold to determine positions, as per the following formula:

$$x_{i,j}^{r+1}=\left\{ \begin{array}{cc} 1, & \text{if }\text{rand}()<\mu \\ 0, & \text{otherwise} \end{array}\right.$$ (27)

where $x_{i,j}^{t+1}$ is the selection status of feature j by wolf i in iteration t+1 , rand() is a random number generated from a uniform distribution, and *μ* is the median of an ascending sequence of random numbers; features greater than *μ* are selected, otherwise, they are not.

**Fig 7:**
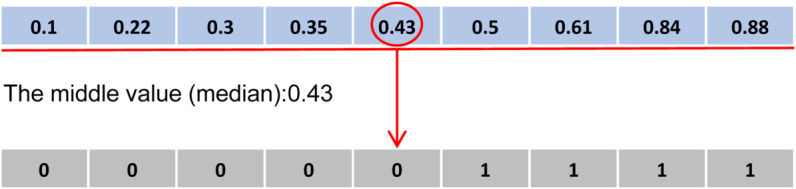
Feature correspondence position selection. This figure is an explanation of Eq (27).

In Fig 7, in the feature selection stage, this study adopts a median based threshold selection strategy. Under this selection mechanism, a feature is selected only when its corresponding value exceeds the threshold. This strategy effectively avoids the extreme cases of not selecting any features or selecting all features, ensuring that the algorithm always obtains a sufficient and high quality feature subset. Secondly, it can adaptively regulate the feature space dimension, reducing the final feature subset dimension. This binarization strategy improves the algorithm’s convergence performance and stabilizes the size of the feature subset.

To overcome the limitations of the GWA algorithm in local development capabilities, we combined the precise development ability of the chaotic dung beetle algorithm with the hierarchical leadership structure of the GWA. The chaotic dung beetle algorithm focuses on and optimizes the top three individuals in the grey wolf population, conducting local development in their vicinity areas. It generates non-periodic search paths through chaotic sequences and adjusts the dung beetles’ movement direction and step size using Eq (22), achieving precise local development. This approach can improve the quality of solutions while maintaining diversity.

**Fig 8:**
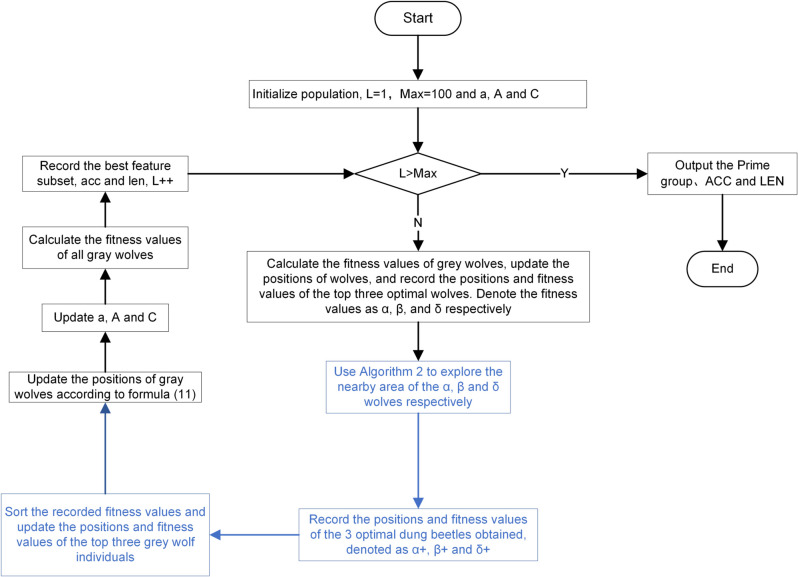
Flowchart of HGW-CDBW algorithm. This flowchart is an introduction to the HGW-CDBW algorithm.

Specifically, we employ the chaotic dung beetle algorithm to perform a search in the vicinity of Alpha, Beta, and Delta wolves, respectively. After three searches, we sort and compare the fitness values of the three best dung beetle individuals with those of the current top three alpha wolves, thereby dynamically updating the positions and fitness values of the top three grey wolf individuals. On this basis, other wolves in the pack are attracted to approach the direction of the optimal solution, and the positions of the wolf pack are adjusted based on the repositioned top three alpha wolves. This approach can preserve the diversity of the solution space. The proposed embedding strategy not only retains the excellent global search capability of the Grey Wolf algorithm but also significantly improves the precision of local searches. Ultimately, a balance between exploration and exploitation is achieved. Fig 8 is a flowchart of the HGW-CDBW; below is the pseudocode:

**Algorithm 3 HGW-CDBW algorithm pseudocode.**



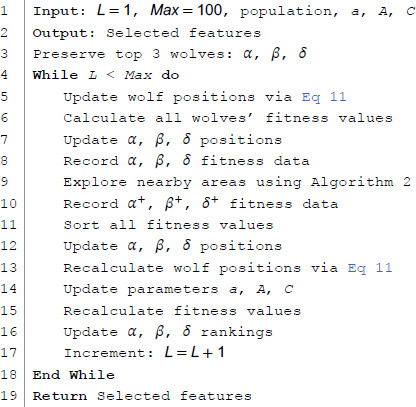



### 3.3 The proposed algorithm

The HMF-W algorithm consists of M1 and M2 modules. In the M1 module, the RF-FIM algorithm mentioned in Sect 2.3 is used for preliminary dimensionality reduction, achieving a significant reduction in dimensions. In the M2 module, the proposed Filter mSMMI and wrapper HGW-CDBW algorithms are used. Through the proposed POM mechanism, we dynamically adjust the execution frequency of Filter and wrapper algorithms to escape local optima. When the wrapper algorithm is executed multiple times and its search effect approaches saturation, the algorithm will execute the Filter algorithm to reinitialize the population. This allows for more effective approaches to the global optimum. Let us now provide a detailed introduction:

#### 3.3.1 Population initalization combining chaos theory.

In swarm intelligence optimization algorithms, population diversity affects the convergence speed and search efficiency. Populations are usually initialized randomly, but this method may lead to uneven population distribution, thus affecting the algorithm’s global search capability and convergence speed. To address this issue, this paper proposes a population initialization method based on chaos theory, enhancing diversity and coverage by mapping chaotic sequences into the search space. By assigning an independent random seed to each individual, the diversity of the initial population is ensured. To initialize the position of each grey wolf using the following formula:

$$P_{ij}=l+(u-l)\cdot \delta$$ (28)

Where, $P_{ij}=p_{i1},p_{i2},...,p_{ij},...,p_{NJ},i=1,2,...,N;j=1,2,...,J.$ where *N* is the number of populations, *J* is the dimension of the search space, *p*_*ij*_ is the value of the *i*-th individual in the *j*-th dimension. $p_{ij}\in [l,u]$, where $l=\min(p_{ij})$, $u=\max(p_{ij})$. *l* and *u* are the lower and upper bounds of the search space, respectively; *δ* is an element from the chaotic sequence, calculated according to Eq (25).

Chaotic systems exhibit better uniformity, sensitivity to initial values, and pseudo-randomness, which help the population to thoroughly explore the solution space. The chaotic sequence is transformed into the problem’s solution space through linear mapping, ensuring the feasibility of the initial solutions. Algorithm 4 demonstrates the specific steps for initializing the population based on chaos theory.

**Algorithm 4 Chaos based population initialization.**



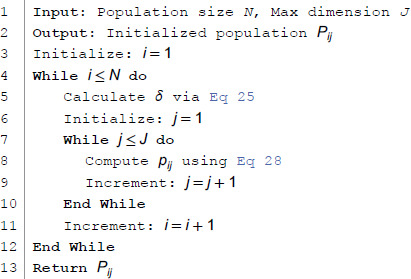



#### 3.3.2 Process optimization mechanism.

In feature selection, the previously mentioned filter and wrapper methods can be combined in various ways to form a hybrid algorithm. This paper introduces the theory of random laser intensity fluctuations, combined with the proposed average improvement rate and mechanism control strategy, forming a POM mechanism that can dynamically adjust the execution frequency of filter and wrapper algorithms. The wrapper algorithm is used for multiple optimizations of the results obtained from the filter algorithm executions.

**Fig 9:**
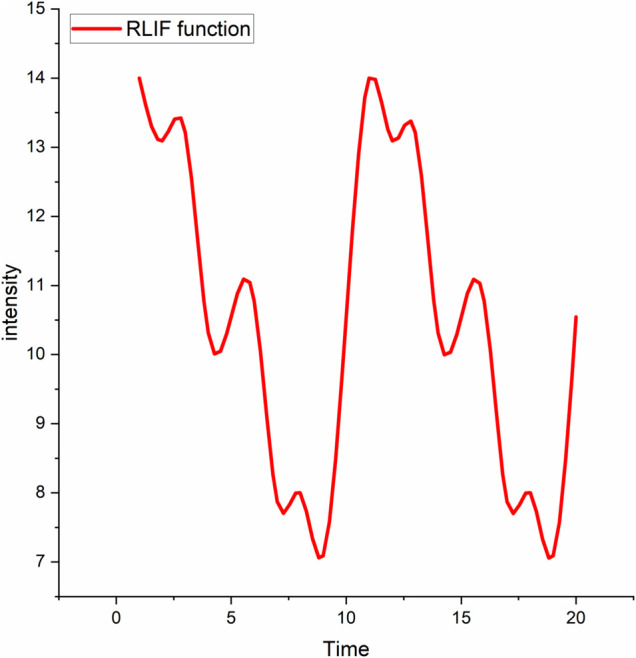
The RLIF function. The Figure demonstrates the variation trend of the random laser intensity fluctuation function.

According to Eq (1), when *A*_1_ = 2.743, *A*_2_ = 1.372, *A*_3_ = 0.914; *f*_1_ = 0.1, *f*_2_ = 0.2, *f*_3_ = 0.4; and $\phi_{1}=\phi_{2}=\phi_{3}=0$, the random laser intensity fluctuation model produces the results shown in Fig 9.

The horizontal axis in the diagram represents time, showing two periods, while the vertical axis represents the intensity of the laser. We can see that there are six extremums in the intensity during each cycle. These six extremums are used to adjust the execution of the filter and wrapper algorithms.

**Average Improvement Rate(AIR)**, as proposed in this paper, is a key performance metric that measures the degree of performance improvement from one iteration to the next. We use it to determine whether the wrapper algorithm is trapped in a local optimum. The specific formula is as follows:

$$\text{AIR}=\frac{\sum_{i=1}^{N-1}\left(\frac{f_{i+1}-f_{i}}{f_{i}}\right)}{N-1}$$ (29)

where, *AIR* is the Average Improvement Rate, *f*_*i*_ and *f*_*i* + 1_ are the fitness scores of the current (*i*^*th*^) and next ((*i* + 1)^*th*^) iterations respectively, *N* is the total number of iterations, and *i* denotes the current iteration number, i=1,2,…,N.

In the early iterations of the HGW-CDBW algorithm, due to the randomness of the initial solutions and the exploration of the solution space, the search effects are more significant, as indicated by a higher AIR value. As the algorithm approaches the optimal solution or saturation point, the improvement in fitness decreases and may even decline. Consequently, the AIR value naturally decreases. The POM mechanism monitors whether the wrapper algorithm’s search results are approaching saturation or getting trapped in local optima by dynamically comparing the count of AIR decrease (CAD) and extrema.

When the CAD value exceeds the current extremum, the algorithm will call the mSMMI filter algorithm again to select a new set of candidate features to reinitialize the population for the HGW-CDBW algorithm, and the CAD value is reset to zero. This achieves the purpose of escaping the local optimum and providing a diverse set of candidate features.

**Fig 10:**
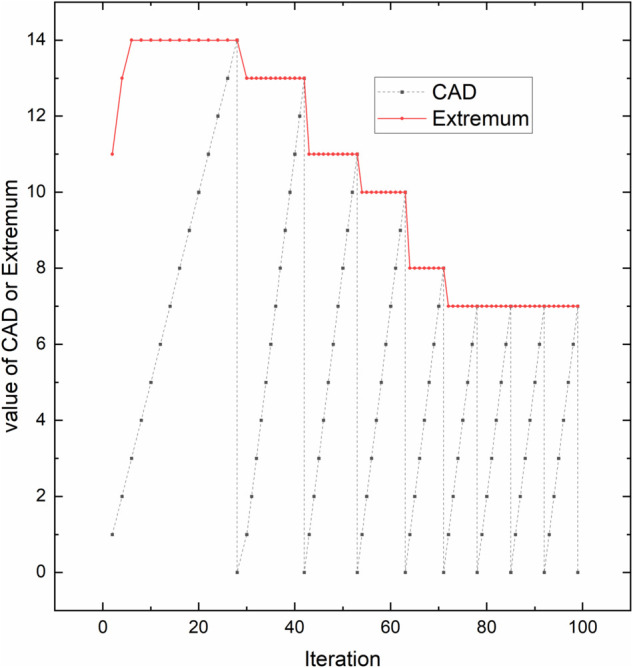
Trend of the values of CAD and Extremum in Colon dataset. The figure demonstrates the variation trends of both the performance metric CAD and the extremum set values during 100 algorithm iterations on the Colon dataset.

**The extremum control (EC)** strategy proposed in this paper is used to regulate the progress of the wrapper algorithm’s search. It dynamically adjusts the current extremum used for comparison by combining the six light intensity extrema mentioned before and the AIR value calculated by Eq (29) to determine the search status of the wrapper algorithm, thus ending the search earlier or later. The specific logic is as follows:

If the AIR value continues to increase, indicating that the search results of the algorithm are stronger than expected, the current extremum is increased. Then the wrapper algorithm will perform more iterations, allowing it to search more solution spaces, making it easier to find the global optimum. If the AIR value continues to decline to zero or even below zero, it indicates that the search effect of the algorithm is weaker than expected. Depending on the extent to which it is below expectations, the current extremum is dynamically decreased to allow the wrapper algorithm to end the current search sooner and revert to the filter algorithm to reinitialize the population. This allows the wrapper algorithm to quickly escape local optima. Fig 10 below shows the changes in the values of CAD and Extremum in the Colon dataset over iterations.

Fig 10 shows that in the early stages of iteration, the search for solutions is ideal, and the EC strategy gradually increases the current extremum, allowing the wrapper algorithm to iterate more times and fully explore the solution space. As the search deepens, the likelihood of finding better solutions increasingly decreases. The EC strategy gradually lowers the extremum to expedite the completion of the wrapper algorithm, which then triggers the bivariate filter algorithm to refresh the population. This achieves the goal of quickly escaping local optima and providing a more diverse set of features. The EC strategy determines when to adjust the process of the algorithm, enabling self-optimization adjustments based on the current search status and historical data, and effectively avoiding premature convergence to local optima.

This paper, based on the theory of random laser intensity fluctuations, combines the proposed average improvement rate and extremum control strategy to form a POM mechanism. Unlike traditional multicollinearity techniques, which mainly focus on handling linear correlations between features, this mechanism can promptly call the proposed bivariate filter algorithm to more effectively help the wrapper algorithm escape local optima. It can also adaptively extend the search process of the wrapper algorithm, improving global search capability.

**Fig 11:**
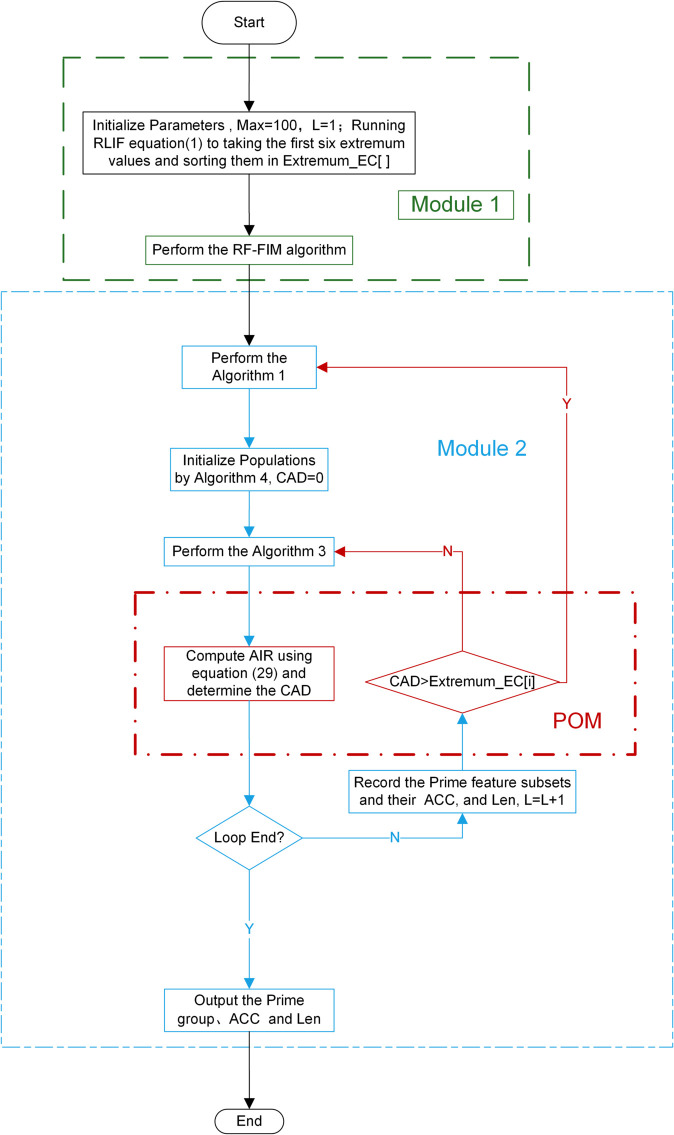
Flowchart of HMF-W algorithm. This figure is an introduction to the proposed algorithm HMF-W.

Below is the pseudocode of the HMF-W algorithm, and Fig 11 is its flowchart. From it, we can see that the HMF-W algorithm first uses the RF-FIM algorithm to reduce the original feature set significantly and obtain the candidate feature subset *F*_1_, as Module M1. Module M2 includes the remaining part of the algorithm, in which Filter Algorithm 1 (mSMMI) is used to perform multi-angle comprehensive consideration on *F*_1_, resulting in the candidate feature subset *F*_*j*_. Then, based on *F*_*j*_, Algorithm 4 is used to initialize the population, which is handed over to Algorithm 3 (HGW-CDBW) for iterative optimization. In addition, the proposed POM is responsible for real-time monitoring of the optimization process of Algorithm 3. When Algorithm 3 gets trapped in a local optimum, it switches to Algorithm 1 to generate a new candidate feature subset *F*_*j*_, which is then given to Algorithm 4 to initialize the population and continue the iterative process of Algorithm 3 until the optimal feature subset is output. Fig 11 below shows the flowchart of the entire HMF-W algorithm:

**Algorithm 5 HMF-W algorithm pseudocode.**



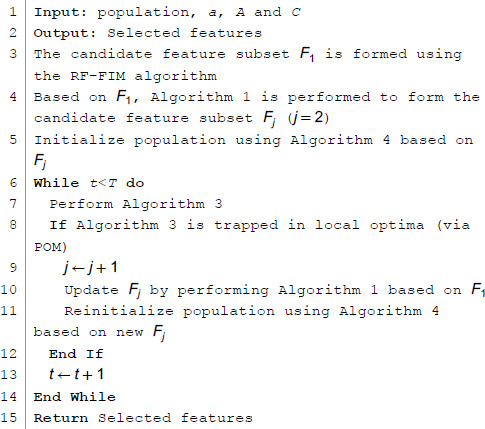



## 4 Experiment and result analysis

In order to fully evaluate the hybrid HMF-W algorithm, this paper conducted several experiments on 10 high-dimensional datasets (DS):

The first group of experiments is an ablation experiment, where the various improved components of the proposed hybrid algorithm are separated out. A total of 7 ablation hybrid algorithms were formed. These include the HMF-W algorithm proposed in this paper, the HMF-W_CDB algorithm (removing the chaotic Dung Beetle algorithm), the HMF-W_GWA algorithm (removing the binary grey wolf algorithm), the HMF-W_M1 algorithm (removing the RF-FIM algorithm or M1 module), the HMF-W_mSMMI algorithm (removing the mSMMI algorithm), the HMF-W_Chaos algorithm (removing chaos theory), and the HMF-W_POM algorithm (removing the process optimization mechanism). Each of these algorithms was tested to validate the effectiveness of each component improvement.

The second group of experiments compares the proposed filter algorithm mSMMI with 10 other classic Filter algorithms, such as ReliefF, Chi-square test, mutual information, normalized mutual information, information gain, Fisher score, Spearman, Pearson, maximum Kendall minimum redundancy, and maximum Kendall minimum chi-square, to demonstrate the excellent performance of the proposed Filter algorithm.

The third group of experiments compares the proposed improved wrapper algorithm HGW-CDBW with other classic wrapper algorithms, such as Ant Lion Optimizer (ALO) [12], Dragonfly Algorithm (BDA) [55], Binary Bat Algorithm (BBA) [56], Genetic Algorithm (GA) [10], Dung Beetle Algorithm (DBA) [14], and Gray Wolf Algorithm (GWO) [43], to validate the effectiveness of the wrapper method improvements.

The fourth group of experiments compares the proposed hybrid algorithm with recent hybrid algorithms to verify the superiority of the proposed algorithm. Although there are many studies on wrapper and filter algorithms, finding an algorithm with exactly the same parameters as the HMF-W algorithm remains a significant challenge. To further validate the algorithm’s performance, we used hybrid algorithms from 2023-2025, such as mRMR-DBA [14], IGMPMMIAPSO [47], TMKMCRIGWO [48], and DFDW [49]. The number of iterations, population size, and the integrated SVM classifier of all the above algorithms are the same as the parameters of the proposed algorithm.

### 4.1 Experimental dataset and algorithm environment settings

#### 4.1.1 Experimental datasets.

To validate the effectiveness of the HMF-W algorithm, 10 high-dimensional datasets from internationally recognized Microarray Data repositories and the Gene Expression Model Selector are used. Table 2 provides a brief description of these datasets, including the dataset name, sample size, number of features, and feature size. The sample size ranges from 62 to 253, the number of features from 2,000 to 24,481, and the number of classes from 2 to 5. Datasets with fewer than 100 features are considered low-dimensional, those with fewer than 2,000 are medium-dimensional, and the rest are high-dimensional. Datasets with two class labels are binary, while those with more than two labels are multi-class.

**Table 2:** Characteristics of the experimental datasets.

No.	Dataset	Classes	Features	Instances	Abbreviation	Data Type
1	Colon Cancer	2	2,000	62	Co	Genome
2	SRBCT	4	2,308	83	SR	Genome
3	Lymphoma	3	4,026	66	Ly	Genome
4	DLBCL	2	5,469	77	DL	Genome
5	Brain_Tumor	5	5,920	90	BT	Genome
6	CLL-SUB-111	3	11,340	111	CS	Genome
7	MLL	3	12,582	72	ML	Genome
8	Ovarian	2	15,154	253	Ov	Proteome
9	SMK-CAN-187	2	19,993	187	SC	Genome
10	Breast	2	24,481	97	Br	Genome

#### 4.1.2 Algorithm parameter settings.

In the next experiments, we compare the proposed HMF-W algorithm with other wrapper and hybrid feature selection algorithms. The SVM classifier uses the radial basis function as the kernel, and its radial basis function and penalty parameters are determined using grid search. Additionally, we used ten-fold cross-validation to compute the classification accuracy (ACC). The dataset is divided into 10 equally sized groups. One group is used as the test set, while the other 9 are combined to form the training set. The average of these classification accuracies and the length of the obtained feature subset (LEN) are used as the result of the fitness function. Based on reference [43], the best classification performance happened when there are 100 iterations and 10 runs. To improve classification performance, this study set the population size to 30 for all algorithms, used 100 iterations, and averaged ACC after testing each dataset 10 times. Table 3 shows the detailed parameter values for each wrapper algorithm.

**Table 3:** Parameter configurations of compared algorithms.

No.	Algorithm	Parameters	Values
1	ALO	*L*, *P*	0, 1, 1.5, 0.5
2	BBA	*Q*_*max*_, *Q*_*min*_	1, 0
3	BDA	*s*, *a*, *c*	0.1, 0.1, 0.7
4	GA	*P*_*c*_, *P*_*m*_	0.7, 0.02
5	CS	*R*, *C*	0.25, 1.5
6	PSO	*C*_1_, *C*_2_	0.9, 0.4, 2, 2
7	GWO	*a*, *r*_1_, *r*_2_	2→0, [0,1], [0,1]
8	CDBA	*s*, *V*, *S*_1_, *S*_2_	3.9, (0,0.2], [0,1], (0,1)
9	mRMR+DBA	*V*, *S*_1_, *S*_2_	(0,0.2], [0,1], (0,1)
10	IGMPMMIAPSO	*P*_*c*_, *P*_*m*_	0.7, 0.5
11	DFDW	*τ*, *b*	-0.3, 1
12	TMKMCRIGWO	*I*_*cc*_, *f*_*cc*_	0.5, 0.01
13	HMF-W	*a*, *r*_1_, *r*_2_, *s*, *u*, *l*	2→0, [0,1], [0,1], 3.9, [–1,–0.3], [0.3,1]

In this study, the Support Vector Machine (SVM) classifier is used as the evaluation criterion for the fitness function. For testing the ACC of the dataset, we used the 10-fold cross validation method. We divide the dataset into ten equal parts using this method, where 9 parts (90%) are merged as the training set, and the remaining part (10%) is used as the test set. This process is repeated 10 times to ensure that every sample is tested. The parameters of the SVM classifier, including the kernel function, penalty parameter, and Radial Basis Function (RBF) kernel parameters, are selected using the Grid Search method. To ensure fairness, except for Sect 4.6, which uses nested cross-validation (i.e., the parameters of the SVM classifier are set within each training fold), the remaining experimental results all use 10-fold cross validation.

#### 4.1.3 Environmental description.

To comprehensively evaluate the performance of the proposed algorithm, we validate the HMF-W algorithm in a CPU environment. Specifically, the environment includes Windows 11, CPU: i5-9400H, 16GB RAM, and an NVMe 1024GB SSD. Through the validation process, we are able to analyze the algorithm’s performance under different conditions and conduct detailed testing and evaluation of its efficiency and ACC. The experiment design is divided into three main modules:

(1). Data Preprocessing Module: The core function of this module is to systematically normalize the raw data in the dataset to ensure that all features are on the same scale, thereby eliminating the impact of different scales on the analysis results. In addition, this module is responsible for extracting basic statistical information from the dataset, including the number of samples and the number of features.

(2). Analysis Module: After completing data preprocessing, we use the HMF-W algorithm to conduct in depth analysis on the normalized data. This algorithm focuses on the relationships between multiple variables and can effectively identify the interdependencies between features and their contributions to the target variable.

(3). Result Output: Finally, the results generated by the analysis module are visualized to verify the ACC and performance of the algorithm.

### 4.2 Ablation experiments

In order to verify the superiority of the dual-module hybrid algorithm HMF-W, the improved components of the algorithm were isolated for processing on the 10 test datasets given in Table 2. A total of 7 ablation hybrid algorithms were formed. These include the HMF-W algorithm proposed in this paper, the HMF-W_CDB algorithm (removing the chaotic Dung Beetle algorithm), the HMF-W_GWA algorithm (removing the binary grey wolf algorithm), the HMF-W_M1 algorithm (removing the RF-FIM algorithm or M1 module), the HMF-W_mSMMI algorithm (removing the mSMMI algorithm), the HMF-W_Chaos algorithm (removing chaos theory), and the HMF-W_POM algorithm (removing the POM mechanism). Each algorithm was run 10 times to get the average ACC, average LEN, and Relative Speedups (RS), as well as the Standard Deviation (SD) of all results. In this paper, the shortest average RunTime (RT) and its lowest SD for each dataset are taken as T and t, respectively, and the comparison results are shown in Table 4.

**Table 4:** Performance comparison of the average ACC (%), LEN, and RS ± SD of 7 ablation hybrid algorithms on 10 datasets.

No.	DS	HMF-W	HMF-W_CDB	HMF-W_GWO	HMF-W_M1	HMF-W_mSMMI	HMF-W_Chaos	HMF-W_POM
		ACC	ACC	ACC	ACC	ACC	ACC	ACC
		LEN	LEN	LEN	LEN	LEN	LEN	LEN
		RS	RS	RS	RS	RS	RS	RS
1	Co	**93.2±1.3**	90.6±0.2	90.8±0.4	86.1±0.7	85.2±1.5	84.6±1.7	65.9±0.1
		**11.3±2.9**	14.0±6.5	30.0±5.4	16.3±0.3	79.9±0.3	35.8±2.0	35.8±7.3
		1.3T±3.3t	2.1T±40.0t	1.1T±1.3t	T±2.0t*	2.2T±6.7t	1.3T±1.7t	1.6T±t
2	SR	**100.0±0**	98.9±1.5	**100.0±0**	**100.0±0**	99.5±0.8	99.6±0.8	95.3±2.1
		**4.4±1.4**	15.4±1.4	27.9±3.6	16.2±2.4	76.6±2.1	38.4±1.1	30.4±6.1
		1.2T±6.0t	1.9T±13.7t	1.2T±22.1t	T±t*	1.5T±5.6t	T±6.7t	1.3T±3.3t
3	Ly	**98.6±0**	**98.6±0**	71.1±0.1	96.9±0.5	95.6±0.8	96.0±0.8	71.0±0.1
		19.1±4.8	21.6±6.6	28.5±3.5	11.4±2.2*	75.6±4.7	36.8±3.2	35.7±9.9
		1.5T±2.1t	1.5T±5.0t	**T±t**	5.8T±10.0t	2.2T±2.6t	3.0T±7.3t	5.0T±3.8t
4	DL	**100.0±0**	97.5±0	99.7±0.8	96.9±0.7	96.1±0.7	96.3±0.7	76.3±0
		**4.8±1.5**	25.4±2.8	11.8±7.5	19.3±3.1	76.2±2.8	37.8±1.5	37.8±6.1
		**T±t**	2.3T±4.8t	1.1T±4.0t	2.0T±t	1.6T±5.8t	1.1T±4.0t	1.2T±5.8t
5	BT	**94.3±2.9**	91.1±0	92.2±0	89.3±0.8	87.6±1.0	88.3±1.3	66.7±0
		16.3±4.2	15.3±7.1	26.1±4.2	**14.7±3.1**	77.6±1.6	37.7±1.9	34.4±6.4
		1.5T±t	3.6T±4.7t	T±1.9t*	1.5T±1.5t	1.5T±4.0t	1.3T±3.5t	1.9T±2.3t
6	CS	**91.7±1.4**	83.0±0.3	90.8±0.6	78.7±1.7	76.2±1.7	77.4±1.6	50.7±0.6
		20.0±4.8	12.0±4.6*	32.4±5.4	13.2±5.1	78.6±2.4	40.0±0	37.2±4.9
		1.2T±4.0t	2.6T±15.2t	**T±2.0t**	1.9T±t	1.7T±4.0t	1.6T±10.6t	1.9T±7.2t
7	ML	**99.2±0.7**	98.2±0.6	98.5±0.4	95.8±1.1	93.9±2.1	94.1±1.3	94.5±2.1
		**9.1±1.8**	15.3±5.7	28.3±2.6	10.7±3.6	76.7±3.2	38.7±1.3	56.7±3.5
		1.4T±3.0t	2.4T±6.3t	T±t*	1.9T±1.8t	1.6T±4.8t	1.4T±8.3t	1.8T±8.5t
8	Ov	**100.0±0**	**100.0±0**	98.8±0.1	99.4±0.2	99.2±0	99.4±0.2	64.2±0
		**3.0±0**	12.3±7.6	29.9±3.0	16.8±5.8	76.4±3.5	37.8±2.6	37.5±6.9
		1.3T±2.1t	T±14.2t	T±t*	1.8T±1.1t	2.8T±22.3t	2.0T±5.7t	2.2T±4.9t
9	SC	**85.7±1.3**	74.6±1.7	83.3±0.5	72.6±0.8	71.3±0.9	71.4±0.8	53.8±0.6
		**11.8±4.4**	14.8±6.2	29.1±4.7	14.7±4.1	74.5±4.8	35.5±3.6	31.8±5.2
		1.1T±3.3t	1.7T±27.3t	T±4.0t*	1.5T±t	1.5T±31.9t	1.6T±16.4t	1.7T±25.0t
10	Br	**73.8±0.5**	54.7±0.6	73.5±0.8	68.1±2.2	67.1±3.5	66.0±3.1	55.4±0.3
		**12.3±2.1**	15.5±7.0	28.3±3.4	19.5±4.5	73.5±5.5	36.5±2.1	36.6±5.9
		1.1T±2.3t	2.3T±36.0t	T±9.5t*	1.6T±t	1.5T±3.5t	1.2T±6.8t	1.3T±1.8t

The best results are highlighted, and statistical significance based on the Wilcoxon test is indicated by an asterisk (*), denoting p < 0.05.

From Table 4, it can be seen that the HMF-W algorithm performs better in classification accuracy on all datasets, but on the Ly dataset, the proposed algorithm reaches the same accuracy as the HMF-W_CDB algorithm. Then, in most datasets, the feature subset length chosen by HMF-W is better than others, except for Ly, BT, and CS datasets, where the proposed algorithm is slightly worse than HMF-W_M1 and HMF-W_CDB. Finally, on the DL dataset, the proposed algorithm’s RS is significantly better than the other algorithm components. On most datasets, the relative speedup of the proposed algorithm is slightly worse than that of the HMF-W_GWO algorithm. Overall, the HMF-W algorithm achieves excellent classification accuracy while selecting a feature subset with lower length, balancing both classification precision and convergence. Moreover, the relative speedup of the proposed algorithm is also relatively better, surpassing at least half of the ablation hybrid algorithms. This shows the proposed algorithm has excellent classification accuracy, strong convergence, and good computational efficiency.

**Fig 12:**
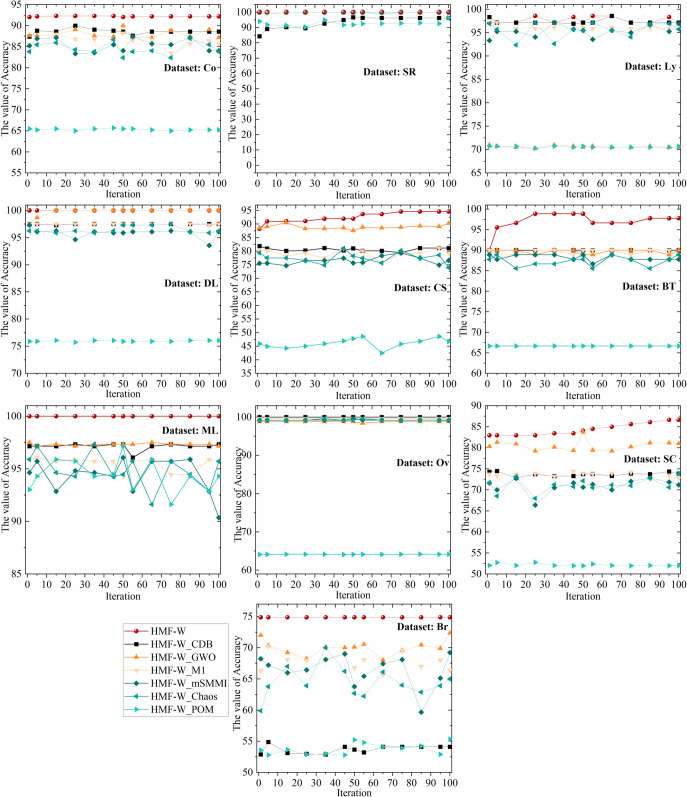
The convergence trends of ACC of the 7 algorithms on 10 Datasets. This figure demonstrates the iterative convergence of classification accuracy of the proposed algorithm and six ablation algorithms on 10 datasets.

**Fig 13:**
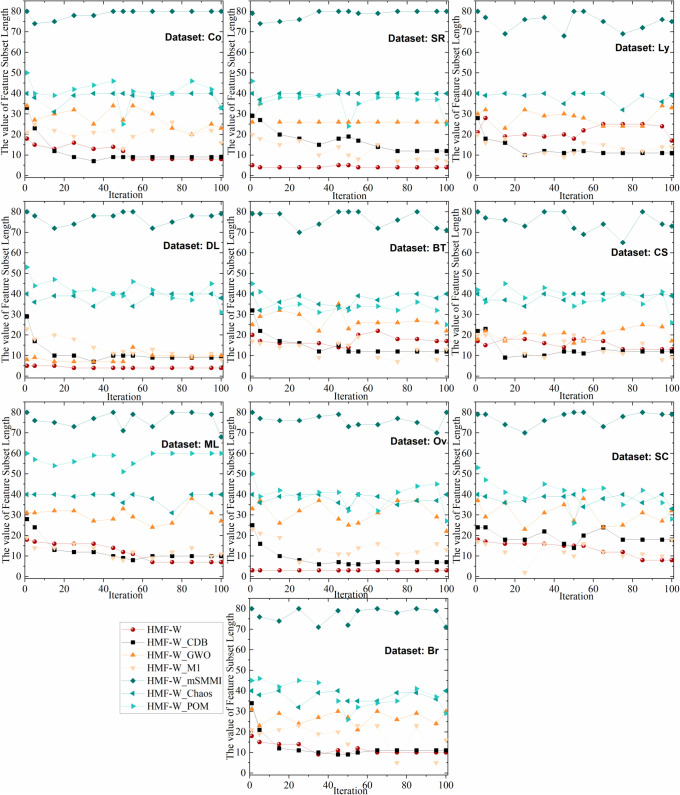
The convergence trends of LEN of the 7 algorithms on 10 Datasets. This figure demonstrates the iterative convergence of feature subset lengths of the proposed algorithm and six ablation algorithms on 10 datasets.

To more comprehensively assess the algorithm’s robustness, Table 5 presents the worst, best, and average ACC of the HMF-W algorithm on 10 datasets, as well as the corresponding feature number selected for the worst and best ACC. Additionally, we plot the convergence of the performance metrics of the 7 algorithms across all datasets, as shown in Figs 12 and 13.

**Table 5:** Experimental results of HMF-W algorithm on 10 datasets.

No.	DS	ACC(LEN)		
		Worst	Best	Avg.
1	Co	92.3(17)	95.3(9)	93.2(11.3)
2	SR	100(6)	100(2)	100(4.4)
3	Ly	98.6(27)	98.6(14)	98.6(19.1)
4	DL	100(9)	100(4)	100(4.8)
5	BT	91.1(21)	98.9(9)	94.3(16.3)
6	CS	90.1(29)	94.6(13)	91.7(20.0)
7	ML	98.7(12)	100(6)	99.2(9.1)
8	Ov	100(3)	100(3)	100(3)
9	SC	84.1(20)	87.7(20)	85.7(11.8)
10	Br	73.3(13)	74.9(13)	73.8(12.3)

By combining Table 4 and Fig 12, it can be observed that the proposed algorithm has a better ACC convergence trend across all datasets compared to other ablation hybrid algorithms. Specifically, on the SR dataset, the ACC convergence trend of the proposed algorithm is similar to that of HMF-W_Chaos, HMF-W_GWA, HMF-W_M1, and HMF-W_mSMMI algorithms, all of which outperform other algorithms significantly. On the DL dataset, the ACC convergence trend of the proposed algorithm is almost identical to that of the HMF-W_GWA algorithm, both significantly outperforming other algorithms. On the Ov dataset, the ACC convergence trend of the proposed algorithm is similar to that of the HMF-W_M1 algorithm, both clearly outperforming other algorithms. Therefore, the improvements in the proposed algorithm clearly enhance its ACC.

From Fig 13, it can be seen that on the Ly, BT, and CS datasets, the LEN convergence trend of the proposed algorithm is slightly inferior to the ablation hybrid HMF-W_CDB and HMF-W_M1 algorithms, but still significantly better than other algorithms. Therefore, the improvements in the proposed algorithm effectively enhance its convergence.

Based on Table 4, Figs 12 and 13, it can be concluded that the proposed process optimization mechanism helps improve the algorithm’s ACC, demonstrating its excellent algorithmic control capability. Additionally, the improvements in the other five algorithm components have also enhanced the algorithm’s classification capability to varying degrees, which proves that the improvements in the components of the proposed HMF-W algorithm are effective.

### 4.3 Filter algorithm comparison experiment

To verify whether the proposed Filter method, mSMMI, performs excellently. On the 10 test datasets given in Table 2, the mSMMI algorithm was compared with 10 other Filter methods by running each one once under conditions of selecting 25, 50, 75, 100, 150, 175, 200, 250, 275, and 300 features. The average ACC, RS, and their SD were obtained. In this paper, the shortest average RT and its lowest SD for each dataset are taken as T and t, respectively, and the comparison results are shown in Table 6.

**Table 6:** Comparison of the average ACC (%) and RS ± SD of 11 filter algorithms on 10 datasets.

No.	DS	mSMMI	MKmR	MKMC	NMI	MI	Spearman	Pearson	ReliefF	ChiSquare	IG	FisherScore
		ACC	ACC	ACC	ACC	ACC	ACC	ACC	ACC	ACC	ACC	ACC
		RS	RS	RS	RS	RS	RS	RS	RS	RS	RS	RS
1	Co	**86.6±2.3**	60.8±5.5	68.7±6.0	85.5±1.2	86.1±1.5	85.9±0.8	86.2±1.0	84.2±2.1	74.1±7.2	85.9±1.0	85.9±1.7
		**T±t**	T±t	T±t	T±t	T±t	T±t	T±t	T±t	T±t	T±t	T±t
2	SR	**99.4±0.8**	97.1±1.3	84.6±6.0	95.3±6.1	99.2±1.4	95.8±6.1	96.7±4.8	99.2±1.2	97.5±2.4	96.7±3.7	99.1±0.8
		**T±2.0t**	T±t	T±t	T±t	T±t	T±t	T±t	T±t	T±t	T±t	T±2.0t
3	Ly	**98.6±1.4**	95.7±4.4	88.7±5.9	97.4±2.0	98.2±1.0	95.8±3.0	97.5±0.7	78.0±6.8	93.9±5.1	90.4±7.7	94.7±6.0
		**T±t**	2.0T±t	T±t	T±t	T±t	T±t	T±t	T±t	T±t	T±t	T±t
4	DL	**96.3±2.8**	92.4±2.0	91.7±1.4	95.8±1.1	95.9±1.6	94.5±3.2	93.3±1.0	93.3±2.3	86.3±6.4	93.2±1.1	95.0±3.0
		**T±t**	T±t	T±t	T±t	T±t	T±t	T±t	T±t	T±t	T±t	T±t
5	BT	86.9±1.9	85.6±3.2	79.3±3.8	81.1±4.3	85.8±1.9	82.8±2.8	81.6±4.7	85.6±3.4	77.3±6.3	80.8±5.5	**88.2±1.2**
		1.5T±t	1.5T±t	1.5T±t	T±t*	1.5T±t	T±t*	1.5T±t	1.5T±t	1.5T±2.0t	1.5T±t	T±t*
6	CS	83.7±1.8	80.5±2.0	75.7±3.4	73.6±5.7	78.8±5.3	79.0±4.3	65.8±13.0	**84.1±2.0**	70.5±4.6	76.0±2.6	75.0±1.8
		**T±t**	1.3T±t	1.3T±t	1.3T±t	1.3T±t	1.3T±t	1.3T±t	1.3T±t	1.3T±t	1.3T±t	1.3T±t
7	ML	**95.8±1.0**	94.8±1.5	87.9±4.0	95.0±1.3	95.7±1.0	93.9±0.9	94.6±2.0	95.6±1.2	79.7±5.6	94.6±1.0	94.5±2.0
		2.0T±t	2.0T±t	2.0T±t	T±t*	2.0T±t	T±t*	T±t*	2.0T±t	2.0T±t	T±t*	T±t*
8	Ov	**99.5±0.2**	94.4±0.7	93.5±0.4	98.6±0.7	99.1±0.2	99.2±0.4	99.2±0.4	99.4±0.5	94.9±1.7	99.2±0.3	99.1±0.4
		**T±t**	1.5T±1.5t	1.8T±1.5t	T±t	T±t	T±1.5t	T±t	T±t	2.0T±2.0t	T±1.5t	T±t
9	SC	**73.7±0.9**	73.6±2.2	72.9±1.4	73.0±1.9	73.6±1.0	72.9±1.1	72.6±5.3	73.4±1.3	72.0±4.8	72.7±1.6	72.5±1.4
		1.2T±t	**T±t**	1.2T±t	**T±t**	1.2T±t	1.2T±t	1.2T±t	1.2T±t	1.2T±t	**T±t**	1.2T±t
10	Br	**76.6±1.1**	**76.6±1.2**	76.5±2.1	76.0±2.1	76.3±1.3	51.0±11.2	53.9±11.6	51.1±10.9	50.1±5.1	76.3±2.1	76.1±1.4
		**T±t**	T±t	T±t	T±t	T±t	T±t	T±t	T±t	T±t	T±t	T±t

The best results are highlighted, and statistical significance based on the Wilcoxon test is indicated by an asterisk (*), denoting p < 0.05.

From Table 6, we can see that, on the Br dataset, MkmR and mSMMI algorithms are comparable. Except for the BT and CS datasets, where mSMMI performs slightly worse than FisherScore and ReliefF, mSMMI outperforms other Filter algorithms on the remaining 8 datasets. This proves that the proposed Filter method has superior classification performance.

Due to the excellent computational cost of filter algorithms, the computational efficiency gap between filter algorithms is difficult to demonstrate. Therefore, in the Wilcoxon test for RS results, only algorithms such as ChiSquare, FisherScore, Relief, MKMC, and mKMR show significant differences with the proposed algorithm on up to 4 datasets. Through comparing RS, we can observe. On the CS dataset, the RS of the mSMMI algorithm is significantly better than the others, reducing the average runtime by 25%. On the Ly and Ov datasets, the proposed algorithm’s RS is on par with most algorithms and better than a few others. On the Co, SR, DL, and Br datasets, the RS of all algorithms is the same. However, on the BT, ML, and SC datasets, the proposed algorithm is on par with other bivariate filter algorithms but slightly worse than most univariate algorithms. It is clear that the proposed bivariate filter method has an advantage in computational efficiency.

To provide a more comprehensive evaluation of the algorithm’s robustness, Table 7 shows the worst, best, and average ACC of the mSMMI Filter algorithm on 10 datasets, as well as the number of selected features corresponding to the worst and best ACC.

**Table 7:** Experimental results of mSMMI algorithm on 10 datasets.

No.	DS	ACC(LEN)		
		Worst	Best	Avg.
1	Co	83.57(25)	90.71(200)	86.6
2	SR	97.50(25)	100.00(50)	99.4
3	Ly	94.29(25)	100.00(200)	98.6
4	DL	89.46(25)	98.75(300)	96.3
5	BT	82.22(25)	88.89(50)	86.9
6	CS	80.15(75)	85.68(100)	83.7
7	ML	94.29(300)	97.32(75)	95.8
8	Ov	99.22(275)	99.62(50)	99.5
9	SC	72.19(250)	74.88(75)	73.7
10	Br	75.22(25)	78.33(75)	76.6

DS represents the datasets.

### 4.4 Wrapper algorithm comparison experiment

To verify the superiority of the proposed algorithm’s wrapper part. On the 10 test datasets given in Table 2, we compared 6 other wrapper algorithms with the wrapper HGW-CDBW algorithm proposed in this paper. To improve computational efficiency, each algorithm randomly selects hundreds features before performing wrapper search. Each algorithm was run ten times to get the average ACC, average LEN, and RS, along with the SD of all results. In this paper, the shortest average RT and its lowest SD for each dataset are taken as T and t, respectively, and the comparison results are shown in Table 8.

**Table 8:** Comparison of the average ACC (%), LEN, and RS ± SD of 7 wrapper algorithms on 10 datasets.

No.	DS	HGW-CDBW	CDBA+SVM	GWA+SVM	ALO+SVM	BBA+SVM	BDA+SVM	GA+SVM
		ACC	ACC	ACC	ACC	ACC	ACC	ACC
		LEN	LEN	LEN	LEN	LEN	LEN	LEN
		RS	RS	RS	RS	RS	RS	RS
1	Co	**85.2±1.5**	65.9±0.2	74.6±0.4	64.8±0	64.8±0	67.5±0.7	64.8±0
		**17.1±2.2**	53.1±5.7	51.9±6.1	39.5±7.1	44.5±4.7	58.1±7.2	42.8±6.0
		1.1T±t	1.3T±4.8t	T±1.2t*	4.0T±17.2t	1.8T±2.8t	T±3.8t	1.5T±2.3t
2	SR	**100.0±0**	95.3±2.7	**100.0±0**	95.1±0.9	99.2±1.4	99.2±0.7	95.9±1.6
		**19.5±1.3**	57.4±2.8	75.4±10.7	55.2±6.3	52.1±5.0	54.1±8.2	54.5±5.0
		**T±t**	2.4T±11.0t	3.9T±9.3t	15.1T±56.0t	9.5T±12.2t	4.4T±63.8t	1.1T±1.9t
3	Ly	**96.1±0.9**	71.0±0.2	71.1±0.2	68.4±0	68.4±0	71.2±0.1	95.8±1.2
		**22.9±0.4**	52.0±6.8	28.8±2.3	44.6±5.6	42.5±4.2	43.5±5.0	48.2±4.7
		**T±1.9t**	T±1.4t*	1.5T±t	5.8T±5.2t	1.8T±2.5t	1.8T±2.3t	3.1T±1.3t
4	DL	**96.0±1.8**	93.2±0	76.3±0	89.3±1.9	93.8±0	76.2±0.1	91.3±1.8
		**22.8±5.2**	57.5±2.8	49.8±2.2	43.6±4.5	44.1±4.2	46.3±6.1	43.7±4.5
		**T±t**	2.5T±8.2t	3.0T±7.2t	13.6T±476.5t	6.2T±9.8t	5.0T±3.0t	1.5T±2.8t
5	BT	**88.1±0.7**	66.7±0.2	66.7±0	68.0±0	68.0±0	66.7±0	81.3±0.9
		**27.2±7.1**	51.1±7.6	55.4±1.8	40.1±3.3	44.3±3.8	48.3±7.5	43.7±3.8
		**T±2.6t**	2.5T±9.6t	3.6T±4.5t	14.4T±24.6t	7.9T±t	7.2T±45.5t	3.4T±2.8t
6	CS	**76.2±2.7**	50.6±1.6	51.6±1.2	56.5±2.1	56.1±1.4	52.1±1.4	62.6±2.2
		**23.2±8.9**	56.7±3.8	31.3±5.8	40.5±5.0	43.0±6.3	50.6±1.6	45.8±3.3
		**T±t**	2.4T±12.4t	4.4T±11.8t	16.9T±164.6t	20.8T±28.2t	20.8T±31.6t	10.9T±16.6t
7	ML	94.0±1.9	**97.2±0**	45.1±0.2	65.2±2.0	62.6±4.5	63.3±4.0	67.3±1.9
		**20.9±6.7**	59.0±1.5	23.8±6.1	44.5±5.1	43.7±6.1	42.1±3.6	48.1±5.1
		**T±t**	2.5T±6.4t	3.7T±3.6t	19.9T±204.0t	15.2T±11.1t	15.2T±5.3t	6.1T±5.0t
8	Ov	**99.3±0.2**	64.3±0.2	75.4±2.6	64.0±0	64.0±0	64.2±0	64.0±0
		**42.8±9.4**	53.8±5.0	75.5±3.1	43.0±5.3	43.5±5.1	43.6±6.1	44.3±5.0
		**T±t**	1.9T±2.9t	5.0T±6.8t	11.7T±37.0t	15.5T±240.8t	15.1T±223.5t	23.1T±135.2t
9	SC	**71.2±1.0**	54.7±0.3	55.1±0.5	51.7±0	51.7±0	53.8±0.5	51.7±0
		**20.6±9.6**	55.8±3.3	36.2±4.6	40.5±2.8	47.5±6.1	50.0±4.1	44.3±3.8
		**T±t**	2.1T±3.3t	4.6T±9.9t	30.4T±50.4t	38.7T±100.7t	39.0T±96.7t	21.3T±102.9t
10	Br	**64.9±2.1**	54.6±0.5	55.3±0.4	53.8±0	53.8±0	55.5±0.7	53.8±0
		38.5±8.0	57.5±3.5	27.3±5.8*	42.9±4.1	40.9±4.9	49.8±5.2	44.5±2.2
		**T±t**	2.5T±7.2t	3.9T±36.2t	31.7T±948.0t	47.8T±896.5t	46.4T±1182.5t	19.8T±19.2t

The best results are highlighted, and statistical significance based on the Wilcoxon test is indicated by an asterisk (*), denoting p < 0.05.

From Table 8, we can see that, on most datasets, the improved algorithm HGW-CDBW shows higher average ACC than other wrapper algorithms. Only on the SR dataset, the improved algorithm is on par with the GWA+SVM algorithm. Also, on the ML dataset, the improved algorithm is slightly worse than the CDBA+SVM algorithm. On most datasets, the improved algorithm’s average LEN is better than other algorithms, except on the Br dataset, where the improved algorithm is slightly worse than the GWA+SVM algorithm. On most datasets, the RS of the improved method is significantly better than other wrapper algorithms. Except on the Co dataset, where the improved method is slightly worse than the GWA+SVM algorithm. On all datasets, the improved algorithm shows statistically significant differences in average ACC, LEN, and RS compared to most other algorithms. This proves that the improved wrapper algorithm in this paper has superior convergence and classification performance.

To provide a more comprehensive evaluation of the algorithm’s robustness, Table 9 shows the worst, best, and average ACC of the HGW-CDBW algorithm on 10 datasets, as well as the corresponding LEN.

**Table 9:** Experimental results of HGW-CDBW algorithm on 10 datasets.

No.	DS	ACC(LEN)		
		Worst	Best	Avg.
1	Co	83.3(36)	87.4(4)	85.2(17.1)
2	SR	97.5(28)	100(5)	100.0(19.5)
3	Ly	93.8(35)	97.1(3)	96.1(22.9)
4	DL	92.3(30)	97.5(14)	96.0(22.8)
5	BT	86.7(34)	88.9(14)	88.1(27.2)
6	CS	69.2(36)	78.3(12)	76.2(23.2)
7	ML	91.6(33)	95.7(12)	94.0(20.9)
8	Ov	99.2(57)	99.6(25)	99.3(42.8)
9	SC	69.5(39)	72.7(10)	71.2(20.6)
10	Br	62.9(46)	69.0(24)	64.9(38.5)

DS represents the datasets.

### 4.5 Hybrid algorithm comparison experiment

To validate the superiority of the proposed HMF-W algorithm, we compare it with four recent hybrid algorithms on the 10 test datasets given in Table 2. Each algorithm was run ten times to get the average ACC, average LEN, and RS, along with the SD of all results. In this paper, the shortest average RT and its lowest SD for each dataset are taken as T and t, respectively, and the comparison results are shown in Table 10.

**Table 10:** Comparison of the average ACC (%), LEN, and RS ± SD of 5 hybrid algorithms on 10 datasets.

No.	DS	HMF-W	TMKMCRIGWO	mRMR+DBO	DFDW	IGMPMMIAPSO
		ACC	ACC	ACC	ACC	ACC
		LEN	LEN	LEN	LEN	LEN
		RS	RS	RS	RS	RS
1	Co	**93.2±1.3**	81.4±1.2	86.4±0.9	91.7±2.5	73.2±1.0
		11.3±2.9	23.1±3.5	63±5.1	6.3±1.6*	44.9±7.0
		**T±1.4t**	3.3T±15.9t	4.0T±t	5.2T±8.4t	4.8T±14.9t
2	SR	**100.0±0**	**100±0**	**100±0**	98.9±0.9	**100±0**
		**4.4±1.4**	23.8±3.0	61.7±5.6	34.1±12.0	53.4±11.6
		**T±4.7t**	2.0T±4.4t	1.8T±t	3.4T±24.0t	2.4T±3.6t
3	Ly	98.6±0	71.1±0.1	87.1±0	100±0*	85.9±0.4
		**19.1±4.8**	22.4±12.5	63.1±3.4	25.9±1.7	62.5±6.0
		**T±1.4t**	2.6T±3.9t	2.9T±t	2.0T±8.2t	2.7T±6.7t
4	DL	**100.0±0**	**100±0**	**100±0**	**100±0**	**100±0**
		**4.8±1.5**	22±5.3	61.5±6.5	34.5±3.7	16.5±2.5
		**T±t**	2.9T±6.8t	3.5T±2.2t	4.5T±17.7t	3.7T±18.5t
5	BT	**94.3±2.9**	67.8±1.5	91.1±0	66.7±0	66.7±0
		**16.3±4.2**	17.8±18.0	60.7±6.0	36.5±18.0	56.6±13.0
		**T±t**	1.9T±3.1t	1.8T±1.9t	2.9T±9.4t	2.2T±2.6t
6	CS	**91.7±1.4**	72.5±1.7	90.4±1.4	84.1±1.7	76.8±2.8
		**20.0±4.8**	22.5±8.8	68.2±4.6	36.9±9.1	22.5±3.5
		**T±3.3t**	2.2T±18.5t	2.1T±t	3.6T±15.7t	2.2T±9.5t
7	ML	**99.2±0.7**	94.1±3.5	98.2±0.4	94.6±0.9	73±0.1
		**9.1±1.8**	12.9±3.0	58±3.5	50±0	70.9±7.1
		**T±1.2t**	1.8T±3.1t	2.2T±t	3.0T±9.4t	1.9T±11.2t
8	Ov	**100.0±0**	90.5±1.3	64.2±0	95.7±0.7	90.3±0.2
		**3.0±0**	23.1±9.4	62.4±4.0	42.9±16.9	74.8±8.7
		1.1T±t	2.6T±3.0t	1.3T±1.8t	1.1T±3.1t	**T±2.8t**
9	SC	**85.7±1.3**	70.4±2.1	83.9±0.9	78.4±0.7	72.7±3.6
		**11.8±6.2**	26.1±10.4	64±4.0	29.3±6.0	48±2.4
		**T±1.6t**	2.7T±18.1t	1.5T±t	1.6T±3.8t	1.8T±11.6t
10	Br	**73.8±0.5**	55.3±0.3	73.5±0.4	68±2.4	56.9±1.0
		**12.3±4.5**	21.2±11.0	66.9±4.9	23.4±10.8	44±5.5
		**T±1.3t**	1.9T±7.6t	2.3T±t	2.8T±19.7t	2.0T±4.6t

The best results are highlighted, wilcoxon test’s statistical significance is indicated by an asterisk (*), denoting p < 0.05.

From Table 10, we can see that the proposed HMF-W algorithm performs much better in accuracy on most datasets, except that it is slightly worse than DFDW on the Ly dataset. Then, its chosen feature subset length is shorter on most datasets, except for Co where it’s slightly worse than DFDW, showing good convergence and classification performance. Next, the proposed algorithm’s RS is significantly better than most other algorithms on most datasets, except on the Ov dataset, where it is slightly worse than the IGMPMMIAPSO algorithm. From a statistical Wilcoxon test perspective, the proposed algorithm’s results show significant differences from most other algorithms across all datasets. Except for the proposed algorithm’s ACC, which does not show significant differences from at least two algorithms on the SR, Br, and DL datasets, the LEN does not show significant differences from two algorithms on the CS and Br datasets, and the RS does not show significant differences from the DFDW and IGMPMMIAPSO algorithms on the Ov dataset. Finally, the proposed algorithm shows a relatively low standard deviation in most datasets, showing its results are robust.

**Fig 14:**
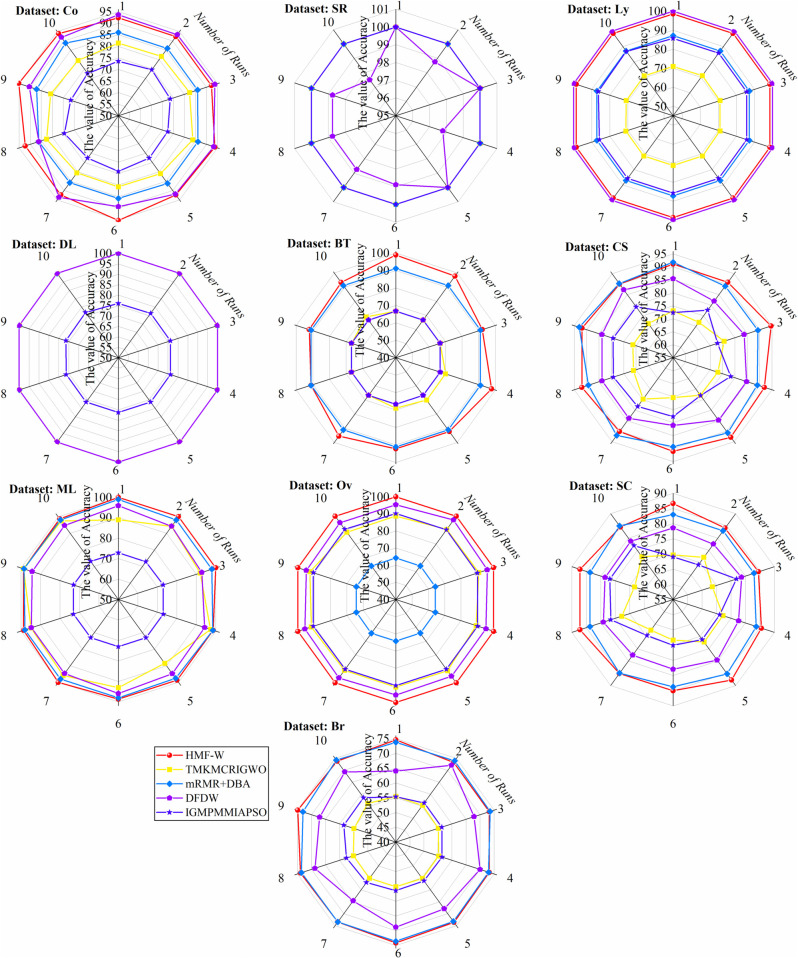
The single ACC values of the 5 algorithms running on 10 datasets. This figure displays the classification accuracy values of 5 hybrid algorithms over 10 executions on 10 datasets.

To further validate the classification performance of the proposed algorithm, Fig 14 shows the ACC values obtained by these five hybrid algorithms on ten datasets. In this figure, the vertical coordinate of the radar chart is the ACC value, and the number at the edge is the number of runs.

From Fig 14, we can see that the proposed HMF-W algorithm performs much better in ACC on most datasets, except that it is slightly worse than DFDW on the Ly dataset. Then, its chosen feature subset length is shorter on most datasets, except for Co where it’s slightly worse than DFDW, showing good convergence and classification performance. Next, the proposed algorithm runs faster than most others, except on Ov where it is slightly slower than IGMPMMIAPSO. Based on the Wilcoxon test, the results show significant differences from most other algorithms on all datasets. Except that its ACC on SR, Br, and DL is not significantly different from at least two algorithms, its subset length differs insignificantly only on CS and Br, and its running time only on Ov compared with DFDW and IGMPMMIAPSO. Finally, the proposed algorithm shows a relatively low standard deviation in most datasets, showing its results are robust.

**Fig 15:**
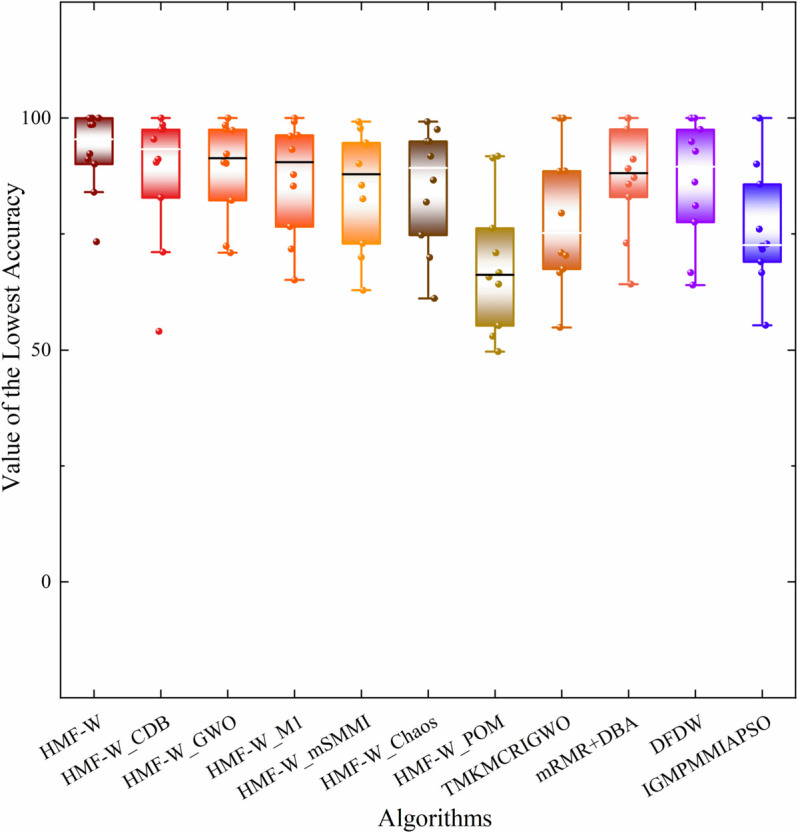
Comparison of the lowest ACC values of 11 algorithms on 10 Datasets. This figure compares the minimum classification accuracy values achieved by 11 hybrid algorithms on all 10 experimental datasets.

**Fig 16:**
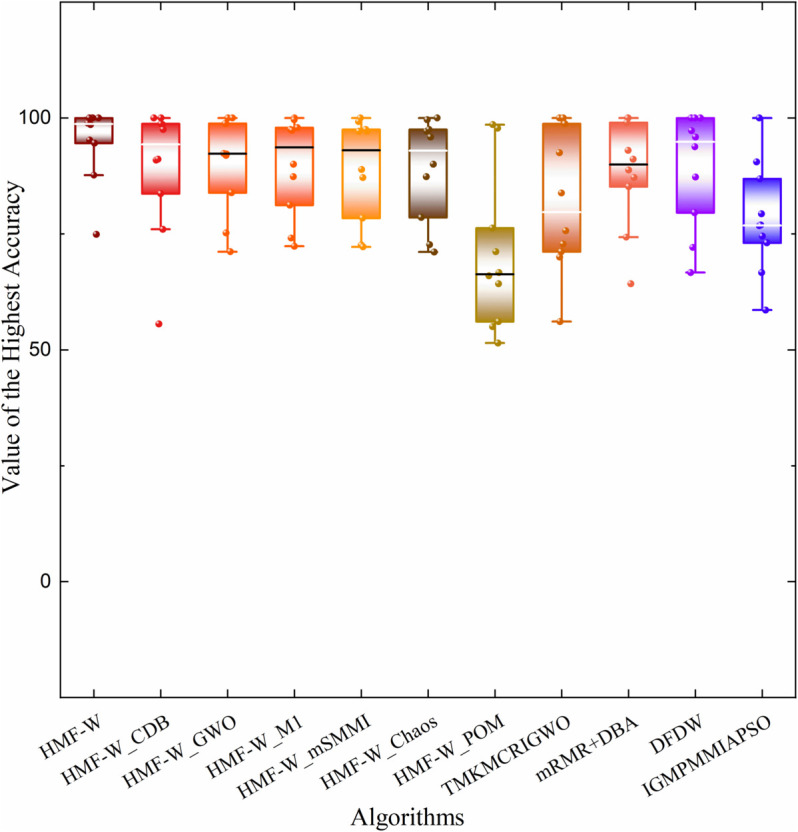
Comparison of the Highest ACC values of 11 algorithms on 10 Datasets. This figure visualizes the maximum classification accuracy values attained by 11 hybrid algorithms across the 10 experimental datasets.

To further describe the differences between HMF-W and the other 10 hybrid algorithms. The minimum and maximum ACC rates obtained by the 11 algorithms combined with the SVM classifier on ten datasets are shown in Figs 15 and 16, where the horizontal axis represents the datasets and the vertical axis represents the ACC values. The points in the figure represent the lowest or highest ACC values.

From Figs 15 and 16. It can be seen that the classification ability of the HMF-W algorithm, compared to other algorithms, is the best across all five percentiles (minimum, quartile (25th percentile), median, quartile (75th percentile), and maximum). Therefore, based on Table 10 and Figs 14 to 16, it can be seen that the HMF-W algorithm has significant advantages in reducing feature dimensions and finding the optimal ACC. The standard deviation of the classification results of the proposed algorithm is relatively low on most datasets, so it can be concluded that the dynamic feature selection strategy used by the filter method is robust. Moreover, this algorithm, with its outstanding search ability, can effectively solve the problems faced during the feature selection process. In summary, this algorithm can balance the goals of ACC and feature count.

### 4.6 Nested cross-validation hybrid algorithms comparison experiment

To further demonstrate the superiority of the proposed algorithm, this section presents new results by applying nested cross-validation to five hybrid algorithms. Specifically, on 10 test datasets, the proposed algorithm was compared with four recent hybrid algorithms. Each algorithm ran 10 times to obtain average ACC, average LEN, RS, and the SD of all results. In this paper, the shortest average RT and its lowest SD for each dataset are taken as T and t, respectively, and the comparison results are shown in Table 11:

**Table 11:** Nested cross-validation comparison of the average ACC (%), LEN, and RS ± SD of 5 hybrid algorithms on 10 datasets.

No.	DS	HMF-W	mRMR+DBO	TMKMCRIGWO	DFDW	IGMPMMIAPSO
		ACC	ACC	ACC	ACC	ACC
		LEN	LEN	LEN	LEN	LEN
		RS	RS	RS	RS	RS
1	Co	90.9±0.5	90.4±1.6	95.7±2.6*	89.7±1.5	89.4±1.7
		**16.0±3.5**	35.3±4.2	40.9±3.8	40.5±16.0	59.9±19.5
		**T±t**	2.4T±1.7t	2.4T±1.2t	3.3T±2.4t	2.3T±2.7t
2	SR	**100.0±0**	93.1±3.7	99.6±0.8	99.7±0.8	98.0±1.7
		**25.0±4.7**	31.7±6.3	40.6±4.8	69.4±9.6	78.5±1.9
		**T±t**	1.4T±1.6t	1.5T±1.1t	2.4T±2.6t	1.9T±2.1t
3	Ly	**100.0±0**	93.4±3.0	100.0±0	100.0±0	97.7±2.2
		**29.0±6.7**	29.8±7.6	39.9±3.5	64.9±14.0	64.0±15.1
		**T±2.2t**	1.6T±1.7t	1.6T±t	2.4T±3.2t	2.0T±2.7t
4	DL	**100.0±0**	94.6±1.7	98.6±1.4	99.8±0.5	96.7±1.8
		**23.3±7.2**	33.7±5.8	42.5±5.3	62.7±10.4	60.3±20.1
		**T±t**	1.5T±2.0t	1.5T±t	2.4T±5.0t	2.1T±5.9t
5	BT	**94.1±0.6**	87.0±2.3	92.6±2.2	88.3±1.1	89.6±1.4
		**18.3±2.5**	27.9±6.0	41.7±4.1	53.7±14.8	74.1±4.8
		**T±t**	1.8T±5.0t	2.0T±5.8t	2.6T±11.2t	1.8T±8.3t
6	CS	**88.3±0.9**	78.2±3.9	86.9±2.5	84.6±3.2	75.6±3.7
		**19.1±4.4**	58.1±6.5	41.4±2.5	31.7±8.4	62.8±12.3
		**T±t**	1.8T±3.1t	1.5T±1.9t	2.1T±1.9t	2.3T±4.8t
7	ML	**100.0±0**	89.4±3.7	98.8±1.1	95.7±1.5	94.8±2.7
		**21.2±3.8**	59.1±5.2	43.0±3.8	45.3±8.3	76.2±6.5
		**T±t**	1.3T±8.0t	T±4.7t	2.1T±6.3t	1.8T±12.4t
8	Ov	**100.0±0**	95.3±1.7	84.8±4.3	80.5±1.9	73.7±2.3
		**16.6±3.8**	30.6±3.4	41.0±3.3	34.9±9.1	61.1±10.4
		1.8T±17.0t	3.1T±6.2t	2.5T±2.9t	T±3.1t	T±t*
9	SC	**84.2±1.3**	75.2±1.2	82.5±3.1	80.9±2.4	74.6±2.2
		**16.7±4.9**	28.9±3.2	41.5±5.0	38.2±9.6	65.8±9.9
		**T±1.4t**	1.8T±1.5t	1.9T±2.0t	1.9T±t	2.3T±1.6t
10	Br	88.5±1.3	74.0±3.3	99.8±0.3*	99.6±0.1	99.8±0.2*
		**26.5±7.5**	28.3±3.2	40.3±3.9	59.4±17.1	63.7±16.2
		**T±t**	1.3T±1.2t	1.6T±1.2t	9.9T±15.9t	9.2T±13.0t

The best results are highlighted, wilcoxon test’s statistical significance is indicated by an asterisk (*), denoting p < 0.05.

As seen in Table 11, all hybrid algorithms experience a significant increase in running time under nested cross-validation, but the proposed HMF-W algorithm remains the best. On most datasets, the proposed algorithm’s average ACC and RS are better than all other hybrid algorithms, except on the Co and Br datasets, where the proposed algorithm’s average ACC is slightly worse than the TMKMCRIGWO, DFDW, and IGMPMMIAPSO algorithms. Also, on the Ov dataset, the proposed algorithm’s RS is slightly worse than the DFDW and IGMPMMIAPSO algorithms. Moreover, by comparing Tables 10 and 11, the average ACC of the proposed algorithm under nested cross-validation is 0.92% higher than that under 10-fold cross-validation. Specifically, on most datasets, the proposed algorithm achieves a higher average ACC under nested cross-validation than under 10-fold cross-validation, with two more datasets achieving 100% average ACC under nested cross-validation compared to 10-fold cross-validation. However, the LEN selected by the proposed algorithm under nested cross-validation is longer, and the running time increases significantly, but it still outperforms the other hybrid algorithms.

### 4.7 Biological genomic explanation

From a biological genomics perspective, only a few microarray genomic data are relatively important for disease diagnosis [54,55]. The proposed algorithm can find the smallest gene subset with the highest ACC. It is crucial to analyze these genes by identifying the obtained genes, as well as their contribution to medical diagnosis and biological significance. Table 12 shows the optimal gene subsets obtained using the proposed algorithm on each dataset.

**Table 12:** The best gene subsets obtained by the proposed algorithm on all datasets.

No.	DS	Index of Features	ACC	TPR	Frequency
1	Co	317,1976,1102,822,1562,948,1494,377,143	95.3	95.3	6
2	SR	338,545,1613,1207	100	100	6
3	Ly	6376,742,955,5593,734,101,4847,1005,908,892,1779,4951,852,2354	98.6	98.5	7
4	DL	3257,52,3942,1127	100	100	6
5	BT	1833,2120,448,2093,2722,2716,5173,1336,3941,309,4794,921,1042,2509	98.9	98.8	5
6	CS	424,3,22,24,44,107,108,131,141,196,201,215,37,232,255,237	94.6	94.6	2
7	ML	1132,7155,7378,6089,7155,7232	100	100	6
8	Ov	2196,183,6781	100	100	6
9	SC	5641,9611,9817,1176,1417,14534,9988,19244,7998,15197,1631,9444, 1040,15485,16075,7573,13101,9876,1585,7952,16436,116,14248,16944	87.7	87.7	5
10	Br	10889,7206,14425,14354,14203,15833,19953,11149,8899,7707,22269, 19893,4729,4691,6214,1409,2769,3232,5044,18811,20859,13510,8449	74.9	74.8	5

As shown in Table 12, the proposed algorithm, when combined with SVM, achieves high ACC and TPR on the optimal gene subset selected for high-dimensional microarray data. Secondly, on most datasets, the proposed algorithm selects the best features at least 5 times in 10 runs. Except for the CS dataset, where the best features were selected only 2 times. The results show that the proposed algorithm has good ability to identify and select optimal features. Finally, the gene features selected by the algorithm have biological significance. We conducted enrichment analysis on the optimal genes selected from the Co, SR, and Ov datasets. Details are as follows:

First, the Co dataset includes 30 cases of colorectal cancer polyps at various stages. It also contains variations in preparation procedures and observations using different surgical instruments during complete colonoscopy. Shieh et al. noted that polyps usually make up a small proportion in complete colonoscopy records [56]. In the 10 runs of the proposed algorithm in this study, the best 9 genes were selected 6 times, with both ACC and TPR being 95.3%. Furthermore, DAVID’s (GOTERM_CC_DIRECT) GO cellular component analysis shows that this gene list is enriched in extracellular exosome functions, with an enrichment score of P=1.95E-2 and Benjamini=9.74E-1.

The SR dataset includes 83 samples, each with 2,308 gene expression values. It covers four types of small round cell blue tumors: 29 cases of Ewing’s sarcoma, 18 of neuroblastoma, 25 of rhabdomyosarcoma, and 11 of Burkitt lymphoma. This is a well-known pediatric tumor classification dataset [59]. In the 10 runs of the proposed algorithm, the best 4 genes were selected 6 times, achieving both an ACC and TPR of 100%. Furthermore, DAVID’s (GOTERM_CC_DIRECT) GO cellular component analysis shows that this gene list is enriched in intracellular membrane bounded organelle functions, with an enrichment score of P=7.00E-3 and Benjamini=0.301.

The Ov dataset consists of surface-enhanced laser desorption ionization time-of-flight protein mass spectrometry data, including 162 ovarian cancer samples and 91 normal tissue samples [23]. The optimal gene subset selected by the proposed algorithm achieved an ACC and TPR of 100%. In the 10 runs of the proposed algorithm, the best 3 genes were selected 6 times, achieving both an ACC and TPR of 100%. Furthermore, DAVID’s (UP_SEQ_FEATURE) annotation enrichment shows that this gene list is enriched in disulfide bond functions, with an enrichment score of P=7.63E-2 and Benjamini=1.14E-1.

The biological interpretation for other biological datasets is as follows:

The Ly dataset includes gene expression profiles for three common adult lymphatic malignancies: 46 cases of diffuse large B-cell lymphoma, 9 of follicular lymphoma, and 11 of chronic lymphocytic leukemia. In 2014, Zhang, Y et al. analyzed certain activities of B cells [57]. The gene subset selected by the proposed algorithm achieved an ACC of 98.6% and a TPR of 98.5%. The DL dataset represents a common type of non-Hodgkin lymphoma, accounting for 30−40% of all such cases. It can be classified into two subtypes based on molecular phenotype: germinal center B-cell-like and activated B-cell-like. In 2017, R.T. et al. studied various types of lymphoma. The ABC subtype has a poor prognosis, with a 5-year survival rate of only 26%, while the GCB subtype has a 62% survival rate [58]. The optimal gene subset selected by the proposed algorithm achieved an ACC and TPR of 100%. The BT dataset includes gene expression profiles for malignant gliomas, recording four types of brain cancer samples: 14 typical brain tumors, 15 lesions, 14 glioblastomas, and 7 anaplastic oligodendrogliomas [2]. The optimal gene subset selected by the proposed algorithm achieved an ACC of 98.9% and a TPR of 98.8%. The CS dataset covers different genetic subtypes of CLL, a mature B lymphocyte malignancy characterized by prolonged lymphocyte survival, immune dysfunction, and treatment resistance [61]. The optimal gene subset selected by the proposed algorithm achieved an ACC and TPR of 94.6%. The ML dataset is a gene microarray for mixed leukemia caused by chromosomal translocation in acute lymphoblastic leukemia. It records three leukemia subtypes: 24 cases of MLL chromosomal translocation leukemia, 20 of acute lymphoblastic leukemia, and 28 of acute myelogenous leukemia [62]. The optimal gene subset selected by the proposed algorithm achieved an ACC and TPR of 100%.

The SC dataset is a Phenome-Wide Association Study related to smoking and cannabis use, involving polygenic risk scores for alcohol, opioids, smoking initiation, and lifetime cannabis use disorders. The optimal gene subset selected by the proposed algorithm achieved an ACC and TPR of 87.7%. The Br dataset includes gene expression profiles from breast cancer patients: 46 cases of recurrent breast cancer and 51 of non-recurrent breast cancer. Due to RNA degradation in the samples [59], the proposed algorithm achieved an ACC of 74.9% and a TPR of 74.8%.

This demonstrates that the proposed HMF-W algorithm can accurately identify and select biologically significant genes in most genomic or proteomic datasets.

### 4.8 Analysis of dimensionality reduction effects

**Fig 17:**
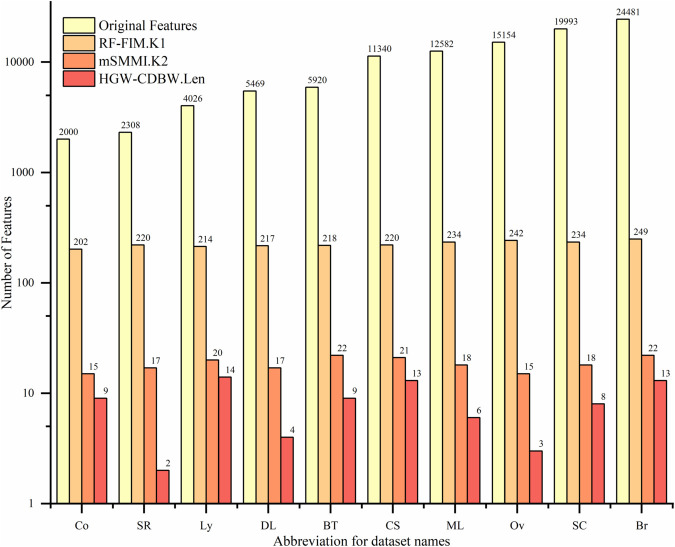
The three-level dimensionality reduction effects of HMF-W on 10 datasets. This figure demonstrates the three-layer dimensionality reduction effects of the HMF-W algorithm on 10 datasets.

**Fig 18:**
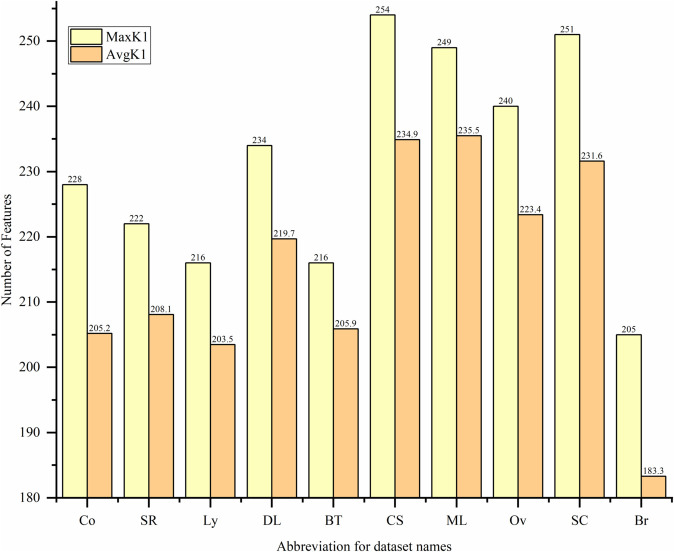
The first-level dimensionality reduction effects of HMF-W on 10 datasets. This figure showcases the primary dimensionality reduction performance of HMF-W during its first-layer processing across 10 datasets.

**Fig 19:**
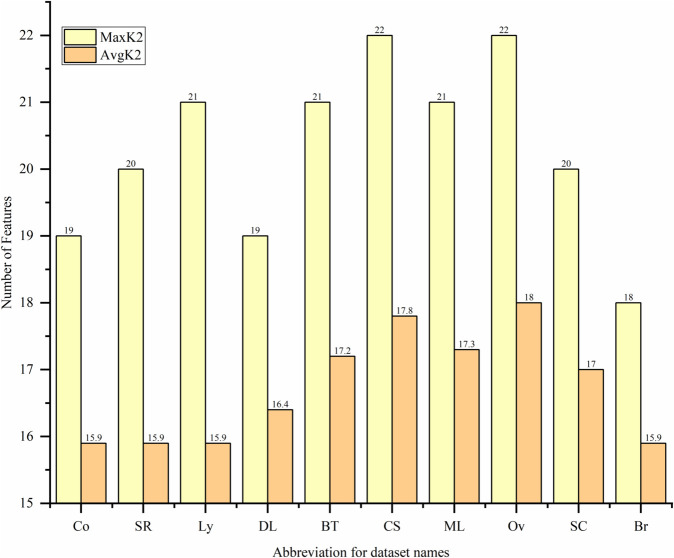
The second-level dimensionality reduction effects of HMF-W on 10 datasets. This figure details the secondary dimensionality reduction results achieved by HMF-W at its second optimization layer over 10 datasets.

The HMF-W algorithm can obtain short feature subsets with high ACC, which is attributed to the algorithm’s good convergence and its dual-module structure design, ensuring the selected feature subsets are shorter. As shown in Figs 17 to 19, the x-axis represents all datasets, while the y-axis represents the number of features.

In Fig 17, we can see that the proposed algorithm achieves excellent dimensionality reduction results on 10 datasets through the dimensionality reduction operations of Modules M1 and M2. The number of features in most datasets drops to single digits. Among them, the Ov dataset shows the most significant effect, reducing from 15,154 features to 3, lowering the dimension to 0.02% of the original. The final LEN of 7 datasets is less than 10. Only the Ly, CS, and Br datasets have a final feature count slightly above 10, with dimensions reduced by at least 0.45% of the original. Therefore, the algorithm has a lower time complexity, with its wrapper part adopting a unique embedded algorithm structure, making its time complexity slightly higher than that of hybrid algorithms such as DFDW, IGMPMMIAPSO, and TMKMCRIGWO.

Figs 18 and 19 display the number of features (K1, K2) after dimensionality reduction by the RF-FIM and mSMMI algorithms, following 10 runs on each dataset. As shown in Fig 18, the average number of features obtained by Module M1 was between 183. 3-245. 3, with all maximum values above 200, and two datasets (CS, SC) exceeding 250. Fig 19 shows that after the execution of the filter algorithm, the average number of features selected by Module M2 ranges from 15.9 to 18, with the maximum values around 20. Among them, three datasets (Co, DL, Br) have fewer than 20 features. The number of selected features can be adjusted in the algorithm’s coding to avoid selection failures. Overall, multiple K values ensure the algorithm can select diverse features, demonstrating the strong convergence of the dimensionality reduction algorithm in Module M2.

### 4.9 Computational complexity

Time complexity is an important metric for measuring the resources (such as time and space) required during algorithm execution. Time complexity and space complexity reflect the time efficiency and memory consumption of the algorithm, respectively. These metrics are used together to evaluate the computational efficiency and performance of the algorithm, providing a basis for comparing different algorithms. The Computational complexity of the 10 hybrid algorithms used for comparison with the proposed HMF-W algorithm is presented in table form, as shown in Table 13.

**Table 13:** Computational complexity comparison of algorithms.

No.	Algorithm	Time Complexity
1	IGMPMMIAPSO	O(T×n×K×S)
2	DFDW	O(T×n×K×S)
3	mRMR+DBA	$O(T\times n^{2}\times S)$
4	TMKMCRIGWO	O(T×n×K×S)
5	HMF-W_POM	O(T×n×K×S)
6	HMF-W_Chaos	O(T×n×(K+3)×S)
7	HMF-W_mSMMI	$O(T\times n^{2}\times S)$
8	HMF-W_M1	O(T×n×K×S)
9	HMF-W_GWA	O(T×n×K×S)
10	HMF-W_CDB	O(T×n×K×S)
11	HMF-W	O(T×n×(K+3)×S)

Where, T represents the total number of iterations, *K* represents the number of selected features, n represents the number of features in the dataset, and S represents the time spent executing the SVM classifier. n×K represents the Computational complexity of updating the algorithm’s position, and K×S represents the Computational complexity of the SVM classifier during one iteration.

In the proposed HMF-W algorithm, RF-FIM and mSMMI algorithms are first used for dual dimensionality reduction on the original dataset. If the original dataset has 2000 features, after the first dimensionality reduction, 200 features may remain, and after the second reduction, 20 features may remain. Then, these 20 features are used as the original feature subset for the wrapper algorithm, which is more beneficial for selecting the most relevant and least redundant features. As the number of features decreases, the computation time also decreases, leading to a corresponding reduction in Computational complexity. In Table 13, since the number of features selected is much smaller than the original number of features in the dataset, and K is much smaller than n, K×n is smaller than n^2^. Therefore, this algorithm has relatively low Computational complexity. Except for the ablation hybrid algorithm without chaos theory, which has nearly the same Computational complexity as the proposed algorithm, the Computational complexity of the proposed algorithm is slightly higher than that of its remaining 5 ablation hybrid algorithms.

Because the wrapper part of the proposed algorithm uses a unique embedded structure, its Computational complexity is slightly higher in theory than hybrid algorithms such as DFDW, IGMPMMIAPSO, and TMKMCRIGWO. However, as shown in Sect 4.5, the proposed algorithm runs faster than most other hybrid algorithms on almost all datasets. This is because we carefully optimized the wrapper process and achieved good results in Relative Speedups. Specifically, the DBO itself is efficient, and we improved its local search ability using chaos theory. Then, the CDBO was applied in the local search phase, where the gray wolf optimizer is least efficient. In this way, the strong classification performance of the gray wolf optimizer is kept, while the computation efficiency is greatly improved.

When dealing with most high-dimensional omics data, the HMF-W algorithm shows higher computing efficiency and faster speed than others, due to its lower complexity and optimized process. This means that within the same time, the proposed algorithm can handle more data or perform more iterations, improving both performance and efficiency. Moreover, its lower Computational complexity shows that the algorithm is more practical for large-scale omics data. Under limited resources, the proposed algorithm may be more suitable for most high-dimensional omics data, as it can run with less memory. However, when handling extremely large omics data, the algorithm might face difficulties due to memory size limits.

## 5 Conclusion

Based on the summary in the introduction, the current research faces three main issues: 1) Univariate Filter methods struggle to break through the theoretical bottleneck of feature interaction compensation, while bivariate Filter methods find it difficult to tackle both the powerful dimensionality curse and limited sample sizes; 2) Existing literature has not fully explored the synergistic framework of dung beetle and grey wolf algorithms; 3) There is limited research on interdisciplinary regulation methods in the field of hybrid feature selection.

To address the above issues and improve local optima, this paper proposes a novel dual-module hybrid feature selection algorithm, HMF-W. The algorithm first uses the RF-FIM algorithm in Module M1 to perform initial dimensionality reduction on the original feature set, and then deeply integrates mSMMI and HGW-CDBW algorithms in Module M2. Through a process optimization mechanism (POM), it allows the wrapper method to backtrack to the Filter method when it falls into a local optimum and updates the population to escape the local optimum, approaching the global optimal solution. Compared to other hybrid algorithms, this algorithm selects shorter-length candidate feature subsets while monitoring the optimization process in real-time through POM, helping the wrapper algorithm escape local optima while avoiding frequent calls to the Filter algorithm.

The proposed dual-module hybrid framework and improvement strategy method is extensively compared with 10 hybrid algorithms from the past three years on 10 public benchmark datasets from MGE. Experimental results indicate that the algorithm outperforms other algorithms, on all datasets, the average classification accuracy is at least 1.3% higher, the average feature subset length is at least 8 units shorter, and the dimension is reduced to less than 0.45% of the original. The results are biologically meaningful and statistically significant.Although the algorithm performs excellently on high-dimensional datasets, its specific optimization for high-dimensional data in the encoding layer makes it challenging to select highly relevant effective features in some low-dimensional datasets.

Future work will expand the algorithm to medium- and low-dimensional datasets, continuously optimizing while maintaining excellent performance on high-dimensional data. Ultimately, the focus will be on solving experimental equipment issues, and if conditions permit, parallel technologies will be adopted to accelerate the computation process, actively verifying the algorithm’s feasibility on ultra-large-scale datasets.
